# Anti-Inflammatory Activity of 1,2-Benzothiazine 1,1-Dioxide Derivatives

**DOI:** 10.3390/ph18101484

**Published:** 2025-10-02

**Authors:** Berenika M. Szczęśniak-Sięga, Izabela Topolska

**Affiliations:** 1 Department of Medicinal Chemistry, Faculty of Pharmacy, Wroclaw Medical University, Borowska 211, 50-556 Wrocław, Poland; 2 Student Science Club of Medicinal Chemistry, Department of Medicinal Chemistry, Faculty of Pharmacy, Wroclaw Medical University, Borowska 211, 50-556 Wrocław, Poland; izabela.topolska@student.umw.edu.pl

**Keywords:** 1,2-benzothiazine, anti-inflammatory activity, COX-1, COX-2, mPGES-1, cytokines, kinases, in vivo studies, in vitro studies

## Abstract

There is an urgent need to develop new anti-inflammatory compounds due to the versatility of their applications and the side effects associated with currently used nonsteroidal anti-inflammatory drugs (NSAIDs). Compounds containing the 1,2-benzothiazine 1,1-dioxide moiety in their structure have demonstrated a broad range of pharmacological activities, among which the anti-inflammatory effect is the most well-documented. Numerous in vivo studies have confirmed the effectiveness of these compounds in alleviating pain and inflammation. In turn, in vitro studies have shown that 1,2-benzothiazine derivatives exhibit anti-inflammatory activity not only through the classical mechanism involving the inhibition of cyclooxygenase (COX) but also through modern, more complex mechanisms. These innovative mechanisms include inhibition of microsomal prostaglandin E_2_ synthase-1 (mPGES-1) or 11β-hydroxysteroid dehydrogenase type 1 (11β-HSD1), suppression of pro-inflammatory cytokines, and modulation of kinase activity involved in inflammatory processes. Importantly, many studies have shown that some new 1,2-benzothiazine 1,1-dioxide derivatives exhibit even stronger anti-inflammatory activity than traditional NSAIDs, making them promising candidates for new drugs targeting inflammation-related diseases. This paper presents a review of 1,2-benzothiazine 1,1-dioxide derivatives investigated for their anti-inflammatory activity in both in vivo and in vitro models, taking into account their various mechanisms of action and potential directions for further research.

## 1. Introduction

**Figure 1:**
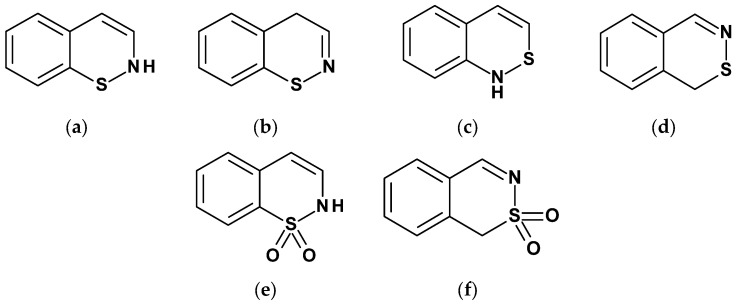
Constitutional isomers of unoxidized (**a**–**d**) and oxidized (**e**,**f**) 1,2-benzothiazine.

**Figure 2:**
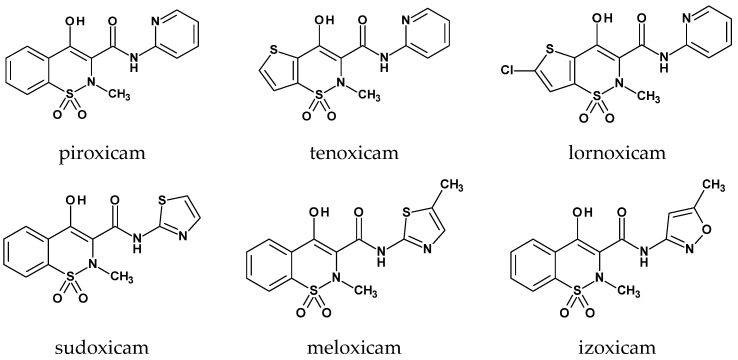
Structures of selected oxicams.

Thiazines belong to six-membered heterocyclic compounds in which two carbon atoms are replaced by two heteroatoms, namely one sulfur atom and one nitrogen atom [1]. Depending on the position of the heteroatoms, these compounds can occur in the form of three isomers: 1,2-thiazine, 1,3-thiazine, or 1,4-thiazine. However, none of the thiazines has been isolated so far [1]. Thiazines gain practical significance when their ring is fused with one or two benzene rings. When the thiazine ring is fused with a single benzene ring, a bicyclic system called benzothiazine is formed. Among the benzothiazines derived from 1,2-thiazine four different systems can be distinguished depending on the position of fusion with the benzene ring. These are: (2H)1,2-benzothiazine (Figure 1a), (4H)1,2-benzothiazine (Figure 1b), 2,1-benzothiazine (Figure 1c), and 2,3-benzothiazine (Figure 1d). Most of these systems appear in the literature as S,S-dioxides. The best-known structure is the 1,1-dioxide of 1,2-benzothiazine (Figure 1e), while the least studied is the 1,1-dioxide of 2,3-benzothiazine (Figure 1f) [2].

The 1,1-dioxide of 1,2-benzothiazine, as a novel heterocyclic compound, was first synthesized by Braun in 1923. However, it was not until 1971, when Lombardino obtained 1,1-dioxo-2*H*-2-methyl-4-hydroxy-*N*-pyridin-2-yl-1,2-benzothiazine-3-carboxamide (now known as piroxicam) that interest in this structure significantly increased. This was due to its strong anti-inflammatory and analgesic properties [3,4]. A few years later, as a result of structural modifications of piroxicam, a new generation of drugs from the oxicam group emerged (including sudoxicam, meloxicam, isoxicam, ampiroxicam, cinnoxicam, droxicam, tenoxicam, and lornoxicam). These compounds exhibited a more favorable pharmacological profile than the parent compound, such as significantly greater specificity toward cyclooxygenase-2 (COX-2) isoform (Figure 2) [5,6,7,8].

As indicated by the literature review, in subsequent years new derivatives of 1,2-benzothiazine 1,1-dioxide were investigated for various pharmacological activities, including antibacterial and antifungal [9,10,11,12,13,14,15], antidiabetic [16,17,18,19,20,21], antiviral [22,23,24], anticancer [25,26,27,28,29], neuroprotective effects [30], as well as enzyme inhibition. Examples of such enzymes include monoamine oxidase [31], alkaline phosphatase [32], calpain [33,34], carbonic anhydrase [35], acetyl- and butyrylcholinesterase [36,37], and urease [38]. Nevertheless, the anti-inflammatory activity of 1,2-benzothiazine derivatives remains the primary focus of interest. Currently used anti-inflammatory and analgesic drugs from the group of nonsteroidal anti-inflammatory drugs (NSAIDs) exhibit numerous side effects. These effects pose a risk to health and life and sometimes lead to discontinuation of treatment [39]. Therefore, the search continues for new, safer anti-inflammatory and analgesic agents for both short-term use and the treatment of chronic inflammatory conditions, such as those seen in rheumatic diseases.

Additionally, the discovery of the molecular mechanisms of carcinogenesis, which indicate a link between cancer and chronic inflammation, has revolutionized the approach to cancer treatment [40]. It has been found that cellular mediators and effectors of inflammation are important components of the local tumor microenvironment. In some cancer types, inflammation is present before neoplastic changes occur. While in others, an oncogenic mutation induces inflammation in the tumor microenvironment, thereby promoting tumor progression. Regardless of the origin of the inflammation, “smoldering” inflammation in the tumor microenvironment exhibits several processes that stimulate cancer development [41]. As a result, anti-inflammatory drugs are gaining importance not only in pain or inflammatory disease management, but also in cancer therapy.

This article presents an overview of 1,2-benzothiazine 1,1-dioxide derivatives studied for their anti-inflammatory effects from the 1990s until 2025. Studies of new compounds are described by division into in vivo and in vitro studies, usually in chronological order. In vivo studies are described first because these studies dominated in the 1990s and early 2000s. Publications describing in vitro studies are newer and are therefore described after in vivo studies. This review demonstrates that in addition to the classical anti-inflammatory mechanism, namely COX-2 inhibition, these compounds also exhibit innovative mechanisms of action, such as inhibition of microsomal prostaglandin E_2_ synthase-1 (mPGES-1), 11β-hydroxysteroid dehydrogenase type 1 (11β-HSD1), or pro-inflammatory kinases and cytokines. New 1,2-benzothiazine 1,1-dioxide derivatives exhibiting these anti-inflammatory mechanisms could potentially be used in the future for the treatment of inflammatory diseases and pain, as well as chemopreventive agents supporting cancer chemotherapy.

## 2. In Vivo Studies

These studies are valuable because they allow for understanding the effects of new chemical compounds within the natural environment of the organism. The goal of preclinical in vivo studies is to assess both efficacy, i.e., whether a new chemical compound exhibits the desired therapeutic properties, and safety, i.e., whether the molecule is non-toxic [42]. However, these studies often do not clarify the mechanism of action of the new substance, thus requiring further in-depth in vitro investigations. Common in vivo tests for anti-inflammatory activity include, among others, the carrageenan-induced paw edema test, the formalin test, and the zymosan test.

The carrageenan test is used to indicate the so-called non-specific inflammatory response of the organism and is conducted on mice or rats [43]. The tested compound is usually administered intraperitoneally to the animals, and after 30 min, a 1% carrageenan solution is injected subplantarly, which induces an acute inflammatory state in the paw. The increase in paw edema is measured several times using a water plethysmometer. The first measurement is taken before the carrageenan injection, followed by three measurements at one-hour intervals after the injection. The severity of the edema in the inflamed paw is assessed by calculating the difference between the paw volume at a specific time after carrageenan administration and the volume measured before its administration.

In the formalin test, pain is induced by a formalin solution injected subplantarly into the mouse paw, which is then observed for 30 min [44]. Injection of diluted formalin causes a characteristic biphasic behavioral response (licking or biting of the paw), referred to as the early and late phases. Phase I of the test (early neurogenic phase) lasts 5 min from the moment of formalin injection and corresponds to the occurrence of acute pain. Phase II (late inflammatory phase) appears 15–30 min after formalin injection and is interpreted as the organism’s inflammatory response. Drugs acting mainly on the central nervous system inhibit both phases (e.g., morphine), whereas drugs acting peripherally inhibit only the late phase (e.g., piroxicam, aspirin).

Zymosan is a polysaccharide of yeast origin that is a strong activator of the immune system. In animal studies, zymosan is often used to model inflammatory conditions such as peritonitis or arthritis [45]. Zymosan stimulates phagocytic cells, such as macrophages and neutrophils, to release cytokines and other inflammatory mediators, the levels of which can be measured. This allows for the assessment of whether a given chemical compound inhibits zymosan-induced inflammation.

**Figure 3:**
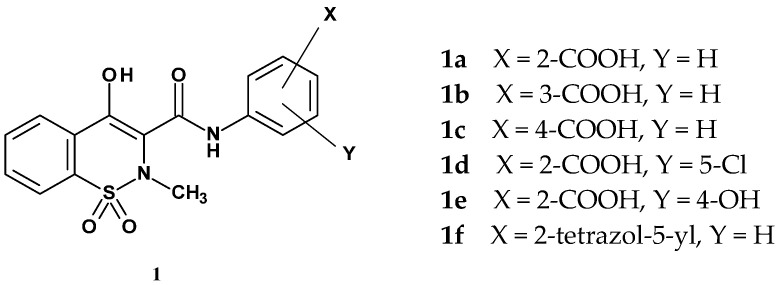
Structures of compounds obtained by Ikeda.

In 1992, Ikeda et al. published the synthesis and studies on the anti-allergic and anti-inflammatory activities of new 1,2-benzothiazine 1,1-dioxide derivatives [43]. The structures of the compounds under investigation were designed to include the 1,1-dioxide 4-hydroxy-2-methyl-1,2-benzothiazine pharmacophore present in piroxicam. To this scaffold, the authors decided to introduce an anthranilamide group, explaining this choice by the presence of this group in tranilast, a drug with anti-allergic properties that specifically inhibits IgE-mediated reactions. As a result of this hybridization, the authors obtained six new compounds with the structure shown in Figure 3. The individual derivatives differed in the position of the carboxyl group on the anthranilic substituent. Compounds **1d** and **1e**, in addition to the carboxyl group on this ring, also contained a second substituent, chlorine or a hydroxyl group, respectively.

Compound **1f** deserves special attention, as it contains a tetrazole ring substituted at the 2-position of the phenyl ring. Ikeda’s team evaluated the obtained compounds for their ability to inhibit histamine release from rat peritoneal exudate cells (PEC) induced by an antigen (egg white). In this test, the compounds were used at a concentration of 200 µM, with piroxicam and tranilast serving as reference drugs. Results are presented in Table 1.

All compounds obtained by Ikeda exhibited inhibitory activity against histamine release. With respect to the position of the carboxyl group on the benzene ring, the *ortho*-substituted derivative (**1a**) was more effective than the corresponding *meta*- and *para*-substituted compounds (**1b** and **1c**, respectively). Among the tested *ortho*-carboxyl compounds, compounds **1d** and **1e**, which contain an additional substituent (chlorine or hydroxyl group) on the phenyl ring, showed stronger activity than compound **1a**. However, the most potent inhibitory effect was observed for compound **1f**, in which the carboxyl group was replaced with a tetrazole ring, suggesting that the tetrazole moiety plays a more significant role in inhibiting histamine release than the carboxyl group. Interestingly, in this test, piroxicam inhibited histamine release more effectively than the antiallergic drug tranilast, which may suggest that anti-inflammatory activity is a highly important factor in the treatment of allergic diseases.

**Figure 4:**
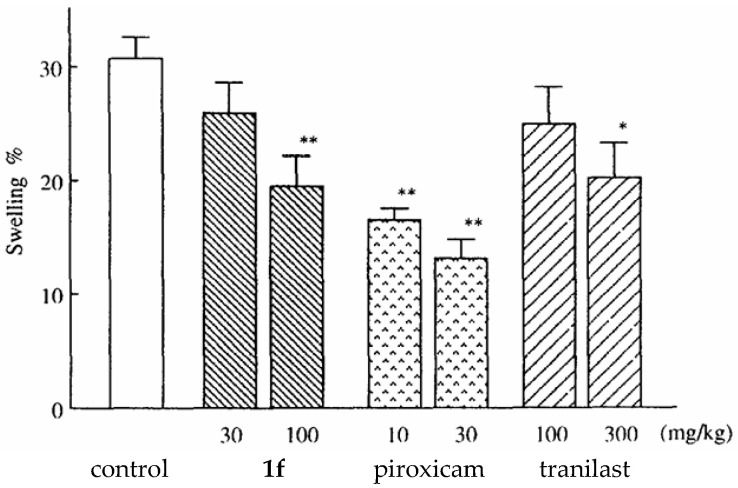
Results of the carrageenan test for compound **1f** and reference compounds. * < 0.05, ** < 0.01.

Two compounds, **1a** and **1f**, were selected for further in-depth in vivo studies. These compounds were evaluated for anti-allergic activity using the passive cutaneous anaphylaxis (PCA) test in rats. The compounds were tested at two doses, 100 and 200 mg/kg, and compared again with tranilast and piroxicam. Compound **1a** showed very weak activity, whereas compound **1f** reduced the PCA reaction in a dose-dependent manner. Its activity was comparable to tranilast but weaker than that of piroxicam. Compound **1f**, being the most active, was subjected to another test, the carrageenan-induced paw edema test, to confirm its anti-inflammatory effect. It was tested at doses of 30 and 100 mg/kg. This study showed that compound **1f** reduced paw edema in a dose-dependent manner, with potency comparable to tranilast but lower than piroxicam (Figure 4).

To determine the mechanism of the anti-inflammatory action of compound **1f**, the authors investigated its effect on prostaglandin synthesis. However, at none of the three tested concentrations (10^−3^, 10^−4^, and 10^−5^ M) did compound **1f** inhibit prostaglandin synthesis, similarly to tranilast. In contrast, piroxicam showed 67% inhibition at a concentration of 10^−3^ M, which may suggest that the anti-inflammatory activity of compound **1f** is based on a different mechanism of action than that of piroxicam and requires further investigation.

**Figure 5:**
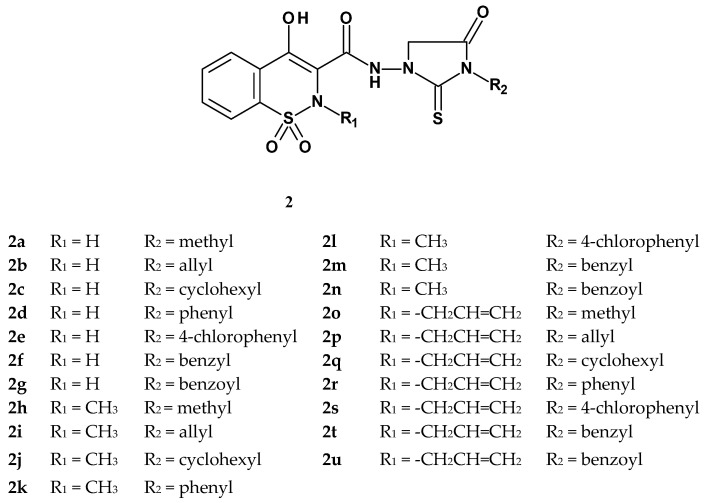
Structures of compounds obtained by Kwon.

Also in 1992, Kwon et al. published the synthesis and evaluation of the anti-inflammatory and analgesic activities of new thiohydantoin derivatives of 1,2-benzothiazine 1,1-dioxide [46]. The authors aimed to modify the structure of piroxicam by replacing the pyridine ring with a thiohydantoin ring substituted with various groups, as many studies had indicated that the heterocyclic ring in the amide side chain significantly enhances anti-inflammatory activity. Additionally, the substituent at position 2 of the piroxicam scaffold was modified either by removing the methyl group or replacing it with an allyl group. Kwon’s team synthesized 21 new 1,2-benzothiazine 1,1-dioxide derivatives, whose structures are shown in Figure 5. The compounds obtained by Kwon featured diverse substituents at the R_2_ position, including both aliphatic groups (methyl or allyl) and cyclic groups (cyclohexyl, phenyl, benzyl, or benzoyl substituents). The R_1_ substituent was either a methyl group, as in piroxicam, an allyl group, or absent.

The compounds were evaluated for anti-inflammatory activity using the carrageenan-induced paw edema test and for analgesic activity using the writhing test. In the carrageenan test the compounds were administered at a dose of 3.3 mg/kg, and in the writhing test at 16.5 mg/kg. Their effects were compared with those of indomethacin and aspirin. The results of these studies are presented in Table 2.

Results of the carrageenan test showed that the most active compounds were those with a methyl group (R_1_ = CH_3_) at the thiazine nitrogen atom, among which the strongest were compounds **2h**–**2l**. In contrast, compounds lacking this group (R_1_ = H) were completely inactive in terms of anti-inflammatory activity (**2a**–**2g**), as were most compounds with an allyl substituent (R_1_ = CH_2_CH=CH_2_), of which only one compound (**2s**) showed moderate activity. Compounds **2h**–**2k** and **2m**, containing a methyl group at position 2 of the thiazine ring, also exhibited very strong analgesic effects in the writhing test, surpassing the efficacy of both indomethacin and aspirin. These results demonstrated the importance of the methyl substituent at the thiazine nitrogen for both anti-inflammatory and analgesic activity.

**Figure 6:**
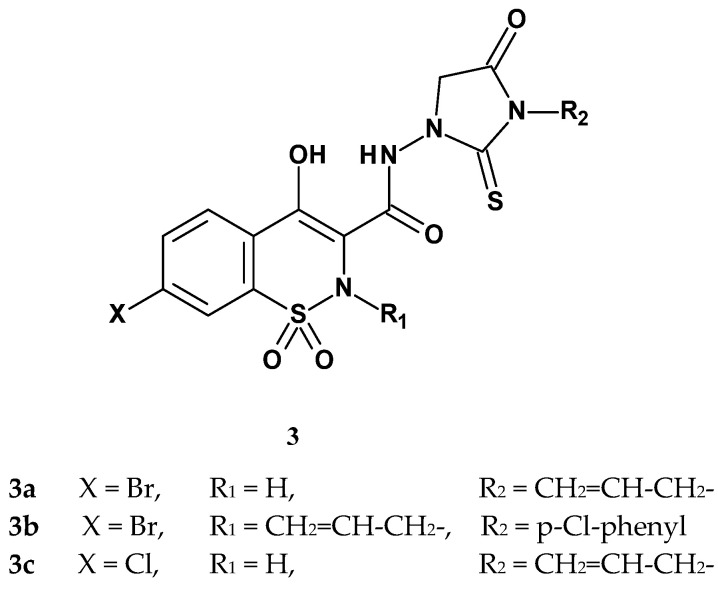
Structures of compounds obtained by Lee.

Continuing Kwon’s research, in 1999 Lee published the synthesis and studies on the anti-inflammatory and analgesic activities of new thiohydantoin derivatives of 1,2-benzothiazine 1,1-dioxide modified by introducing a halogen atom (Cl, Br) into the benzene ring of 1,2-benzothiazine [47]. This resulted in three compounds (Figure 6). Each of the three new derivatives also contained an allyl substituent, differing only in its position, either at R_1_ or R_2_.

The obtained compounds were tested for analgesic activity using the Randall–Selitto method, for anti-inflammatory activity in the carrageenan test, and for gastrotoxicity. Results of these studies are presented in Table 3. In the analgesic study using the Randall–Selitto method, the compounds were administered orally at a dose of 5 mg/kg, and the pain threshold was measured after 30 and 60 min. Results showed a significant increase in the pain threshold 30 min after administration of compound **3c** and piroxicam. A similar effect was observed 60 min after administration of compound **3b** and piroxicam. Compound **3a**, however, did not cause a significant change in the pain threshold. Therefore, the ED_50_ value was determined only for compounds **3b** and **3c**. Compound **3c** exhibited analgesic activity nearly 3.4 times stronger than ibuprofen and 2.3 times stronger than compound **3b**. However, compared to piroxicam, its activity was 6.7 times lower. All tested compounds showed higher efficacy 30 min after administration than after 60 min.

The anti-inflammatory activity was also assessed in the carrageenan-induced edema test at a dose of 5 mg/kg. The edema volume was measured four times (at 1, 2, 3, and 4 h after compound administration). Unfortunately, none of the tested compounds demonstrated anti-inflammatory activity compared to piroxicam, which exhibited very strong anti-inflammatory effects. The final study aimed to assess the degree of gastric mucosal damage caused by the new compounds. The doses used in this test were 1.5 times higher than the ED_50_ values of the compounds. The gastric mucosal damage indices for compounds **3b**, **3c**, ibuprofen, and piroxicam were estimated at 2.9, 2.8, 6.4, and 2.0, respectively. This indicates that compounds **3b** and **3c** cause slightly more irritation than piroxicam but significantly less than ibuprofen. In summary, based on all of Lee’s studies, compound **3c** showed the strongest analgesic effect, being more effective than ibuprofen and causing fewer adverse effects. Nevertheless, its activity was lower than that of piroxicam.

**Figure 7:**
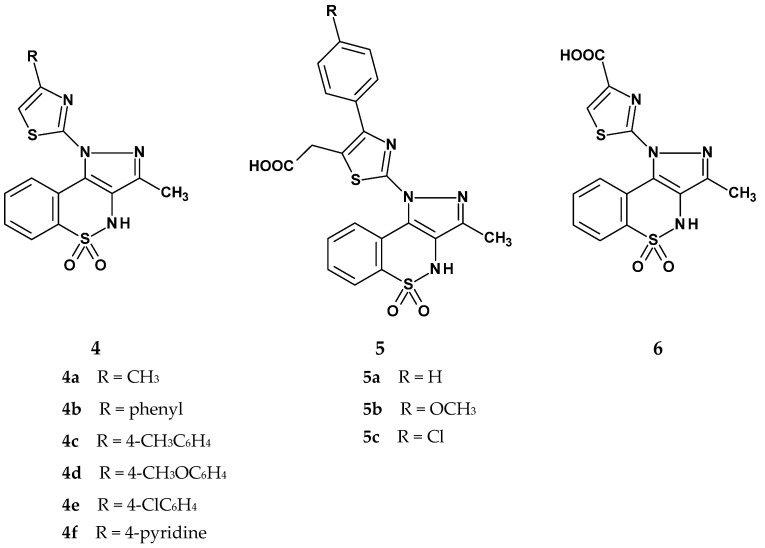
Structures of compounds obtained by Sharma.

Another group of new 1,2-benzothiazine 1,1-dioxide derivatives was synthesized and tested for anti-inflammatory activity by Sharma et al. [48]. When designing these compounds, the authors based their approach on the premise that 1,2-benzothiazine derivatives exhibit anti-inflammatory effects, similar to thiazole derivatives. Therefore, the Sharma team decided to synthesize compounds containing both of these groups within a single molecule. The structures of the newly obtained derivatives are shown in Figure 7. Sharma’s team synthesized three series of novel tricyclic 1,2-benzothiazine derivatives with a thiazole ring introduced at position 1 of the tricyclic scaffold. The first series included six compounds (**4a**–**4f**), which differed in the type of substituent at position 4 of the thiazole ring. In most of these compounds, the R substituent was an aromatic ring, except for compound **4a**. The second series consisted of three compounds whose structures differed from those in the first series by the presence of an additional methylcarboxyl substituent at position 5 of the thiazole ring. The third series included only one compound, which had a carboxyl group at position 4 of the thiazole ring.

All synthesized compounds were evaluated for their anti-inflammatory activity using the carrageenan-induced paw edema test. The compounds were administered via two routes: oral (p.o.) and intraperitoneal (i.p.), at a dose of 100 mg/kg. Ibuprofen was used as the reference drug. The results are presented in Table 4. It was found that the route of administration played a significant role, as most compounds exhibited markedly stronger anti-inflammatory activity when administered intraperitoneally compared to oral administration.

The study results showed that the anti-inflammatory activity generally decreased over time (2 h vs. 3.5 h), which may indicate rapid metabolism of the compounds. Derivatives from series **5** and **6** exhibited stronger anti-inflammatory effects after oral administration compared to series **4**. However, their inhibition values did not exceed that of ibuprofen (62%). The highest anti-inflammatory activity after oral administration was observed for compound **5b**, which contained an *ortho*-methoxy substituent and an additional methylcarboxyl group at position 5 of the thiazole ring (41% inhibition). In contrast, after intraperitoneal administration, some compounds (**4e** and **5a**) showed anti-inflammatory activity comparable to ibuprofen (65% and 66%, respectively, vs. 70% for ibuprofen).

Compound **5a** was the most potent. However, considering its rapid metabolic degradation, compound **4e** appears to be a more promising candidate. Its effectiveness decreased by only 1% after 1.5 h (from 65% to 64%), whereas for compound **5a**, the decrease was as high as 20% (from 66% to 46%).

**Figure 8:**
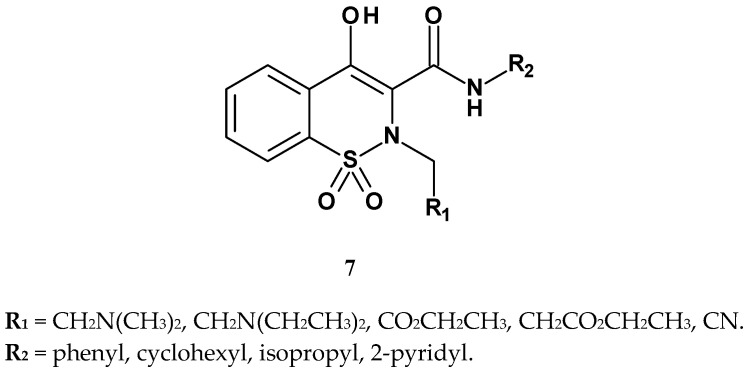
Structures of compounds **7** obtained by Maso.

In the search for new and more effective nonsteroidal analgesic and anti-inflammatory drugs, Maso et al. synthesized oxicams analogs in which the methyl group at the thiazine nitrogen atom was replaced with 2-dialkylaminoethyl or alkyl ester substituents, yielding compounds with the general structure 7 (Figure 8) [49].

The obtained derivatives were evaluated for analgesic activity using the writhing test, and for anti-inflammatory activity using the carrageenan-induced paw edema test. Piroxicam was used as the reference drug. The results for selected compounds are presented in Table 5. In the writhing assay, the most potent compounds were **7c** and **7d**, both containing an alkyl ester substituent at position 2. These compounds were two to three times more active than those with 2-dialkylaminoethyl or cyanomethyl substituents (**7b** and **7e**). Compounds bearing cyclohexyl or isopropyl groups at the R_2_ position showed a significant decrease in analgesic activity (e.g., compound **7k**). However, even the most active compound (**7c**) was over ten times less potent than piroxicam in terms of analgesic effect (ID_50_ = 6.07 mg/kg vs. ID_50_ = 0.76 mg/kg).

In the carrageenan assay, the ester derivatives (**7c** and **7d**) again proved to be the most potent, with activity comparable to that of the reference drug piroxicam. However, compounds **7b** and **7e** exhibited only slightly weaker anti-inflammatory activity. Therefore, the modifications made to classical oxicam structures were beneficial for anti-inflammatory activity but not particularly favorable for analgesic activity.

**Figure 9:**
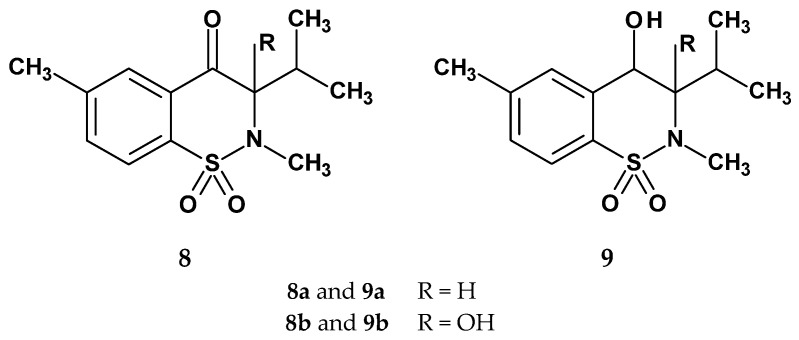
Structures of compounds **8** and **9** obtained by Kacem.

Two additional series of novel 1,2-benzothiazine 1,1-dioxide derivatives, this time lacking the 3-carboxamide moiety present in the structure of oxicams, were proposed by Kacem et al. in 2002 (Figure 9) [50]. Their approach was based on the principle that introducing various modifications to an active structure can influence and improve its biological activity [51].

The authors used the carrageenan-induced paw edema test to evaluate anti-inflammatory activity. The ulcerogenic effect was assessed using the method described by Kulkarni [52]. In both cases, piroxicam served as the reference drug. The compounds were administered intraperitoneally at three doses: 5, 10, and 20 mg/kg. Results for the 5 mg/kg dose are presented in Table 6. In the carrageenan test, the 4-hydroxy derivatives (series **9**) proved to be slightly more active than their keto analogs (series **8**), which the authors attribute to the lower lipophilicity of the hydroxy-substituted compounds.

The most potent anti-inflammatory compound, with an effect comparable to that of piroxicam, was the 4-hydroxy derivative **9b**. The authors also conducted tests on compounds lacking the methyl substituent at the thiazine nitrogen atom to assess its influence on anti-inflammatory activity. These compounds were found to be almost twice as weak, once again highlighting the importance of this substituent for anti-inflammatory efficacy. The ulcerogenicity test revealed that all compounds, at a dose of 10 mg/kg, were almost devoid of ulcerogenic effects, supporting the authors’ claim that the absence of the 3-carboxamide group leads to piroxicam analogs that retain anti-inflammatory activity while protecting the gastric mucosa from damage (tested compounds: UI = 1.1–1.4 vs. UI for piroxicam = 6.2). These findings point to new directions for structural modifications of oxicams, aimed at developing anti-inflammatory agents with reduced ulcerogenic potential.

**Figure 10:**
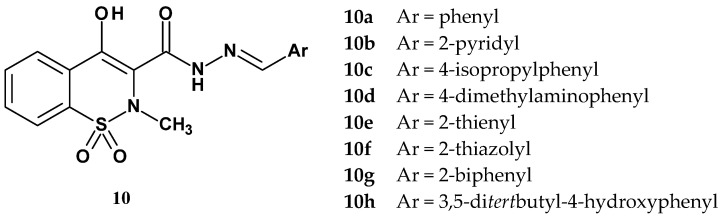
Structures of compounds **10** obtained by Miranda.

In 2012, Miranda and colleagues published the synthesis and evaluation of new *N*-acylhydrazone derivatives of 1,2-benzothiazine 1,1-dioxide for their anti-inflammatory and antinociceptive activities [53]. The target compounds were designed to contain the 1,1-dioxide 4-hydroxy-1,2-benzothiazine pharmacophore, which is present in oxicams. The *N*-acylhydrazone (NAH) structure was recognized as a privileged scaffold, since many such derivatives have been reported in the scientific literature as potent anti-inflammatory and antinociceptive agents. The structures of the new derivatives synthesized by Miranda are shown in Figure 10.

The nature of the aryl substituent (Ar) attached to the imine group varied widely, from unsubstituted or substituted phenyl rings, biphenyl, to pyridine, thiophene, or thiazole rings (**10a**–**h**). The compounds synthesized by Miranda’s team were then evaluated for their analgesic and anti-inflammatory activities using the writhing test, the formalin test, and zymosan- or carrageenan-induced peritonitis tests. In all tests, the compounds were administered at a dose of 100 µmol/kg. Based on these tests, the two most active compounds (**10b** and **10f**) were selected for further evaluation of their inhibitory activity against COX-1 and COX-2 at a concentration of 10 μM. Results of all pharmacological tests and COX inhibition of the new *N*-acylhydrazone derivatives of 1,2-benzothiazine 1,1-dioxide are presented in Table 7.

According to the studies, all compounds synthesized by Miranda’s team (**10a**–**h**) demonstrated analgesic and anti-inflammatory effects in vivo at a dose of 100 µmol/kg. Moreover, these studies showed that the new compounds have a better pharmacological profile than the reference drug piroxicam. However, compounds **10b** and **10f**, which were the strongest in the in vivo tests, did not exhibit inhibitory activity against COX-1 or COX-2. This may suggest that they have a different mechanism of anti-inflammatory activity than classical NSAIDs. This finding requires further, more detailed investigation, especially since compound **10f** appears to be a particularly promising structure, as it proved significantly more potent than the reference piroxicam in three tests (carrageenan, formalin, and zymosan).

**Figure 11:**
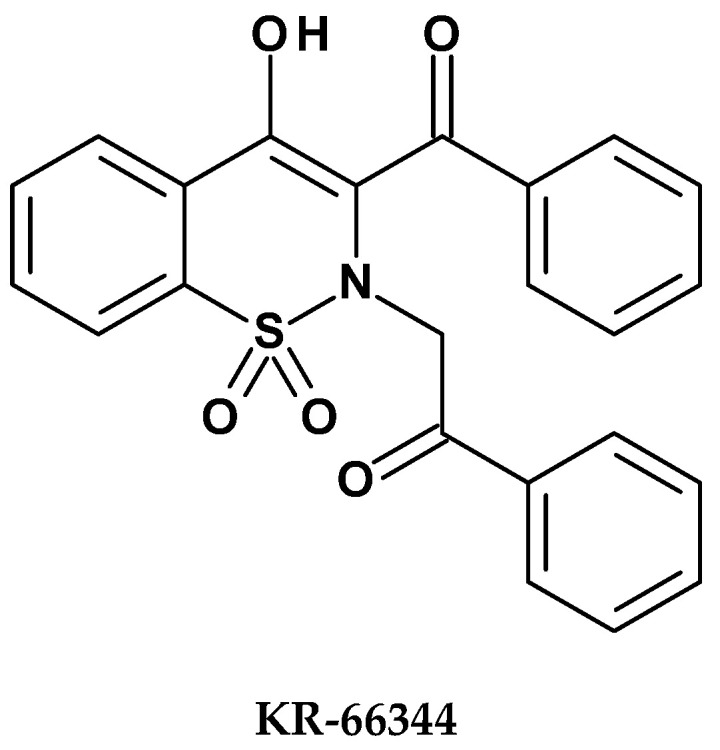
Structure of the 1,2-benzothiazine derivative (**KR-66344**) studied by Park.

In 2016, Park et al. published a study evaluating the anti-inflammatory properties of a 1,2-benzothiazine derivative designated **KR-66344**, which is a selective inhibitor of 11β-hydroxysteroid dehydrogenase type 1 (11β-HSD1), [54]. This enzyme is involved in the conversion of inactive cortisone into active cortisol. The authors justified the purpose of their research by noting that cortisol, as a stress hormone, plays a significant role in modulating the inflammatory response; therefore, the new 1,2-benzothiazine derivative may exhibit anti-inflammatory activity. The structure of the new derivative is shown in Figure 11.

To assess the anti-inflammatory effects of **KR-66344**, the authors conducted a series of experiments analyzing its impact: on the induction of pro-inflammatory cytokines, its effect on the production of nitrites and reactive oxygen species (ROS), as well as its influence on the expression of heme oxygenase-1 (HO-1). Additionally, they examined the effect of **KR-66344** on signaling pathways activated by lipopolysaccharide (LPS). LPS increased 11β-HSD1 activity and expression in macrophages, which was inhibited by **KR-66344**. The authors also evaluated the anti-inflammatory effect of **KR-66344** by silencing 11β-HSD1 in macrophages and assessed the impact of the new derivative on LPS-induced cell death, spleen damage, and mouse survival. To evaluate the activity of **KR-66344**, the studies used carbenoxolone (CBX), a non-selective inhibitor of 11β-HSD1, and ZnPP, a competitive inhibitor of HO-1. Both compounds served as reference substances.

The studies showed that the compound **KR-66344** reduced the production of pro-inflammatory cytokines (IL-1β and IL-6) in the spleens of mice treated with LPS. At a dose of 30 mg/kg, it also inhibited the formation of reactive oxygen species (ROS) and the expression of the enzymes iNOS and COX-2. Furthermore, it was demonstrated that **KR-66344** at a concentration of 10 µM increased the mRNA expression of heme oxygenase-1 (HO-1), which may contribute to enhanced cellular protection against inflammation. The compound also reduced cell death and spleen damage induced by LPS, this effect was observed after administration of **KR-66344** at a dose of 30 mg/kg. LPS significantly increased the phosphorylation of MAPK and NFκB-p65 in spleen cells; however, this effect was completely abolished after treatment with **KR-66344**. Results confirmed the anti-inflammatory activity of the new 1,2-benzothiazine derivative in the mouse model through the mechanisms described above. It can therefore be concluded that selective 11β-HSD1 inhibitors may represent a promising strategy for the treatment of inflammation-related diseases in the future.

**Figure 12:**
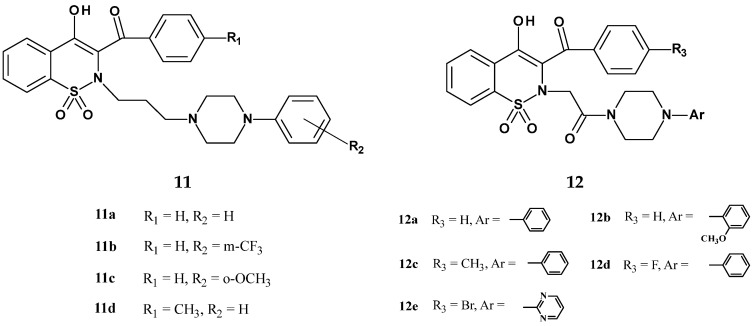
Structures of compounds **11** and **12** obtained by Szczęśniak-Sięga.

In 2019, Szczęśniak-Sięga et al. published the synthesis and evaluation of new oxicam derivatives containing an arylpiperazine moiety with analgesic and anti-inflammatory properties [44]. The team decided to modify the structure of piroxicam due to its strong anti-inflammatory activity and long half-life. In the new derivatives, they introduced a bulky arylpiperazine substituent at position 2 of the 1,2-benzothiazine ring, because Hatnapure noted that the high electron-donating ability of the piperazine moiety can be explicitly correlated with high anti-inflammatory activity of compounds [55]. Additionally, a benzoyl group was introduced at position 3 of the 1,2-benzothiazine ring, as this modification was shown to enhance analgesic activity [56]. The new compounds were divided into two series (**11** and **12**), based on the type of linker connecting the arylpiperazine with the 1,2-benzothiazine (Figure 12).

Series **11** had a propylene linker, which is present in many potent analgesic derivatives of 1,2-benzothiazine. In contrast, the compounds of series **12** contained a 2-oxoethylene linker, because according to the pharmacophore model proposed by Dogruer, the presence of a carbonyl group in the alkyl chain enhances analgesic activity [57]. All obtained compounds were tested for analgesic activity in the formalin test, with piroxicam used as the reference drug. The test results are presented in Table 8.

According to these studies, most compounds showed strong inhibitory effects in both the early (neurogenic) and late (inflammatory) phases of pain, unlike piroxicam, which mainly acts in the late phase. In the second phase, the ED_50_ values for the tested compounds ranged from 4.01 to 9.03 mg/kg, while for piroxicam it was ED_50_ = 18.85 mg/kg. This means that the new derivatives exhibited 2–5 times stronger analgesic activity compared to piroxicam. The authors attributed this effect to the presence of the pharmacophoric arylpiperazine group.

A slightly higher activity was also observed in the late (inflammatory) phase of pain for the compounds from series **11**, which may suggest that the propylene linker between the arylpiperazine and the 1,2-benzothiazine ring is more favorable for the analgesic effect than the 2-oxoethylene linker. Compound **11d** showed the strongest analgesic activity in the late phase, which may indicate its peripheral mechanism of action. The authors associate this strong activity with the presence of a methyl substituent with electron-donating character in the benzoyl group. On the other hand, in the early phase, the strongest analgesic effect was shown by compound **11a**.

It is also worth emphasizing that compounds **11c**, **11d**, and **12b** showed no activity in the early phase of pain, yet they were still more active than piroxicam in the late phase. The high activity of all tested compounds in the late (inflammatory) phase of the formalin test indicates their anti-inflammatory effect. The compounds were also tested for sedative effects due to the appearance of a central mechanism of action (inhibition of the early phase of pain). The results showed that both series **11** and **12** (except for **12d**) do not have sedative effects at the doses used in the formalin test. The sedative effect appeared only at higher doses. The compounds were also evaluated for neurotoxicity using the rotarod test. This test demonstrated that the tested compounds do not cause dysfunction of the central nervous system. Additionally, an acute toxicity study showed that the obtained compounds have a wide safety margin and are significantly less toxic than piroxicam. The ulcerogenic effects of three selected compounds (**12c**, **12d**, and **12e**) was also assessed. Results showed that none of the tested compounds had ulcerogenic activity, in contrast to model piroxicam. Therefore, the studies by the Szczęśniak-Sięga team indicate a very favorable impact of the arylpiperazine group introduced at position 2 of the thiazine ring and provide a basis for further in-depth studies of these derivatives.

## 3. In Vitro Studies

In vitro studies allow for more precise and controlled analysis of biological processes, enabling the determination of drug mechanisms of action or the toxicity of tested substances [58]. This chapter will discuss in vitro studies focused on the anti-inflammatory activity of new 1,2-benzothiazine 1,1-dioxide derivatives. These studies include tests for inhibition of enzyme activities such as cyclooxygenase (COX-1 and COX-2), microsomal prostaglandin E_2_ synthase-1 (mPGES-1), mitogen-activated protein kinases (MAPK), as well as tests evaluating the inhibition of pro-inflammatory cytokine secretion.

### 3.1. Inhibition of Cyclooxygenase (COX)

Cyclooxygenase (EC. 1.14.99.1, prostaglandin H synthase, PGHS) is an enzyme belonging to the family of oxidoreductases, located in the lumen of the endoplasmic reticulum, the Golgi apparatus, and the nuclear membrane of the cell. COX catalyzes the synthesis of prostanoids, i.e., prostaglandins, prostacyclin, and thromboxane from arachidonic acid [59]. To date, three isoforms of this enzyme have been identified: COX-1, COX-2, and COX-3 [60].

COX-1 is a glycoprotein that is present under physiological conditions (the so-called constitutive form) in many tissues such as the kidneys, stomach, intestines, ovaries, and platelets, where it performs numerous protective functions [61].

**Scheme 1:**
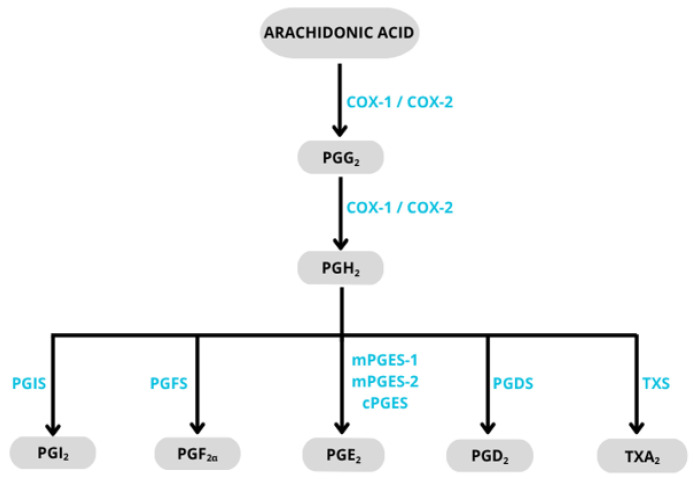
The biosynthetic pathway of prostanoid formation from arachidonic acid. COX-1/COX-2 = cyclooxygenase 1/2; PGIS = prostaglandin I_2_ (prostacyclin) synthase; PGFS = prostaglandin F synthase; mPGES-1/mPGES-2 = microsomal prostaglandin E synthase-1/2; cPGES = cytosolic prostaglandin E synthase; PGDS = prostaglandin D synthase; TXS = thromboxane synthase.

COX-2 is an inducible form of cyclooxygenase, triggered by various factors such as cytokines (IL-1β, IL-6, TNFα), mitogens, or growth factors during inflammation, pain response, tissue injury, or carcinogenesis. Additionally, this form of COX is produced in significant amounts during adaptive processes, such as at the site of wound or ulcer healing. In recent years, a constitutive form of COX-2 has also been identified in certain organs, including the spinal cord, kidneys, vascular endothelium, and uterus [61]. COX-2 has two main functions: cyclooxygenase activity, which oxidizes arachidonic acid to prostaglandin G_2_ (PGG_2_), and peroxidase activity, which reduces PGG_2_ to prostaglandin H_2_ (PGH_2_). Subsequently, various synthases (specific isomerases) convert PGH_2_ into prostaglandins: D_2_ (PGD_2_), F_2α_ (PGF_2α_), E_2_ (PGE_2_), I_2_ (PGI_2_, prostacyclin), and thromboxane A_2_ (TXA_2_), (Scheme 1), [62]. COX-2 is also involved in the metabolism of various chemical compounds, such as those found in cigarette smoke, converting them into highly reactive mutagens that can bind to DNA [63]. Overexpression of COX-2 has been detected in many cancers, and this has been correlated with poorer patient prognosis [64].

The COX-3 isoform has been identified as a “spliced” variant of COX-1 and is mainly present in the brain and spinal cord [65,66,67].

In chronic inflammation and carcinogenesis, the overexpression of inducible COX-2, associated with increased production of PGE_2_, plays a key role [64]. PGE_2_ affects cell proliferation, cell death, and tumor invasion by acting through various membrane receptors called EP receptors (EP1–EP4) [68]. Numerous studies have shown that many tumors (e.g., colon, lung, and breast cancers) produce significantly higher amounts of PGE_2_ compared to the normal tissues from which they originate [69,70,71]. Studies on the pro-carcinogenic mechanism of COX-2 have shown that it is involved in cancer development at various stages, from tumor initiation to the terminal phase of metastasis formation [64]. Targeting COX-2, in addition to its strong anti-inflammatory effect, is one of the newest therapeutic approaches in the treatment of many types of cancers [72].

Searching for selective COX-2 inhibitors based on the structure of the preferential COX-2 inhibitor from the oxicam group (meloxicam) the Lazer’s team decided to perform structural modifications of this drug to obtain derivatives with even greater selectivity toward COX-2. In 1997, they published the synthesis and studies of three series of new meloxicam analogs [73]. The new derivatives were divided into three series according to the site of modification of the reference drug (Figure 2).

**Figure 13:**
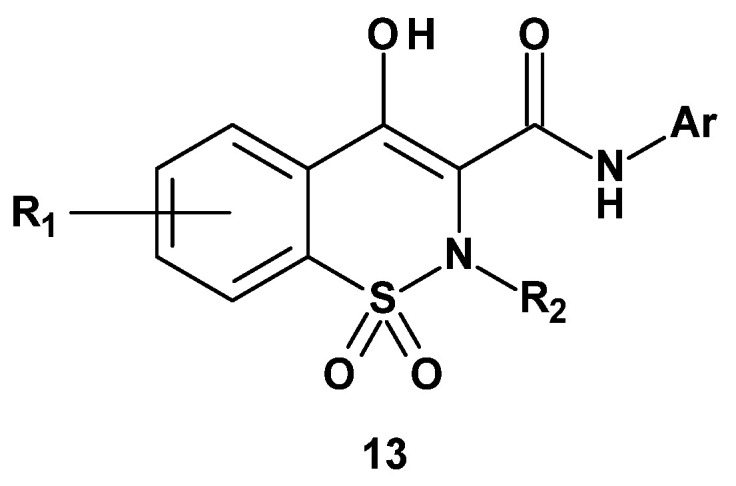
General structure of meloxicam analogs series I obtained by Lazer.

**Figure 14:**
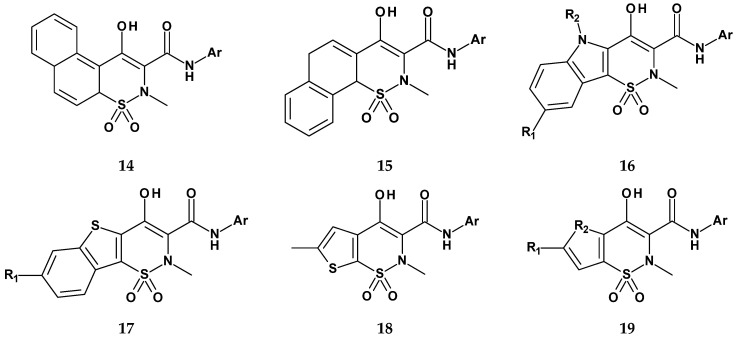
General structures of meloxicam analogs series II obtained by Lazer.

**Figure 15:**
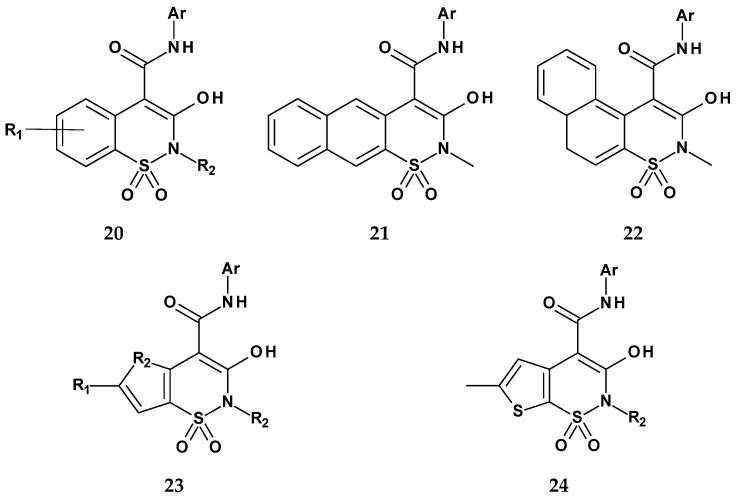
General structures of meloxicam analogs series III obtained by Lazer.

In the first series, substituents at positions 2 and 3 of the thiazine ring and in the benzene ring were modified (Figure 13). In the second series, the benzene ring in the 1,2-benzothiazine was replaced with other aromatic or heteroaromatic rings (Figure 14). In the third series, a carboxamide group was introduced into the thiazine ring at position 4 (Figure 15).

In the first series of derivatives (**13**), 41 new compounds were obtained and subsequently tested for COX-1 and COX-2 inhibition at concentrations of 0.1, 1.0, and 10 µg/ml. Table 9 presents the inhibition results of both isoenzymes for selected compounds from this group at a concentration of 10 µg/mL.

Results of the compounds from series I show that the intended increase in meloxicam’s selectivity toward COX-2 was not achieved through the structural modifications carried out. The importance of the methyl substituent at position 5 of the thiazole ring can be assessed by comparing the COX inhibition by meloxicam and compounds **13a**, **13b**, and **13c**. Meloxicam, which has a methyl group at position 5 of the thiazole ring, inhibits COX-2 by 77%. Removal of this substituent (**13a**) or its replacement with another, such as ethyl (**13b**) or phenyl (**13c**), leads to a significant decrease in COX-2 inhibitory activity—down to 35%, 1%, and 19%, respectively. This series also investigated the effect of replacing the thiazole ring with other rings. This modification can be assessed by comparing meloxicam with compounds **13d** (where a thiadiazole ring was introduced), **13e** (oxazole), **13f** (isoxazole), and **13g** (phenyl). All of these compounds showed reduced COX-2 inhibition, highlighting the importance of the thiazole ring in COX-2 inhibition. Additionally, the effect of substituents in the benzene ring on COX inhibition was examined. Compounds tested in this regard (**13h**–**13l**) showed moderate inhibition (ranging from 40% to 76%) but did not exceed the activity of meloxicam.

In the second series of derivatives, in which the benzene ring in the 1,2-benzothiazine structure was modified by replacing it with another aromatic or heteroaromatic ring, 30 new compounds were obtained. Their general structures are shown in Figure 14. Results of studies on selected compounds from this group at a concentration of 10 µg/mL are presented in Table 10. Most of the compounds from the second series of meloxicam analogs exhibited weak COX-2 inhibitory activity, with the exception of compound **19**, which inhibited the activity of this isoenzyme by 77% and showed approximately 40-fold selectivity toward COX-2. As in the previous series, the applied modifications, such as increasing the molecular size by adding an additional ring or replacing the benzene ring with another, did not enhance COX-2 selectivity.

In the third series of meloxicam derivatives, in which the carboxamide group was shifted from position 3 to position 4 of the thiazine ring, 36 new compounds were synthesized. Their structures are shown in Figure 15. Compounds from series III, like those from the previous series, were tested for COX-1 and COX-2 inhibition at three concentrations: 0.1, 1.0, and 10 µg/mL. Results for selected derivatives from this group at a concentration of 10 µg/mL are presented in Table 11.

The effect of the structural modification in this series differed from the previous ones in two ways. Firstly, phenyl and substituted phenyl amides exhibited very strong COX-inhibitory activity. Compounds **20a**–**20c** were among the most potent COX-2 inhibitors tested by Lazer’s team, showing 97–100% inhibition. However, they also proved to be strong COX-1 inhibitors, resulting in low selectivity toward COX-2. Secondly, compounds with ethyl, cyclopropyl, and benzyl substituents (**20d**–**20f**) also showed strong inhibitory activity against both COX-1 and COX-2. Lazer’s team also synthesized meloxicam derivatives in which the thiazine ring was replaced with other ring systems, such as pyridine or thiopyran. However, these compounds also did not exhibit greater selectivity toward COX-2 compared to the reference compound meloxicam. In summarizing their research, Lazer concluded that this class of compounds may require more extensive structural modifications in order to obtain derivatives with improved COX-2 selectivity.

**Figure 16:**
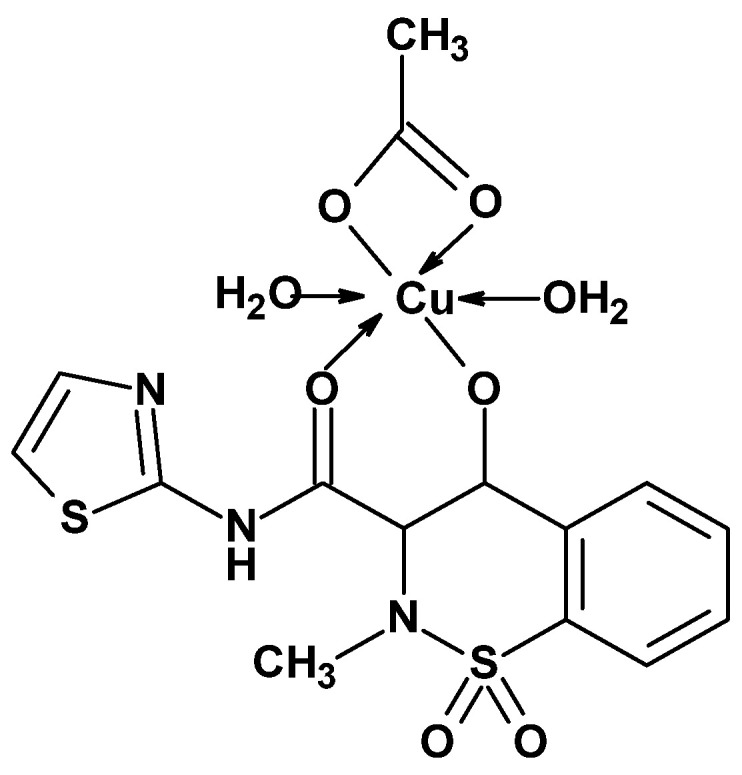
The copper complex of sudoxicam [Cu(sudox)(OAc)(H_2_O)_2_] synthesized by Sherif.

In 2012, Sherif’s team published the synthesis and anti-inflammatory activity studies of sudoxicam (see Figure 2) and its copper complex (Figure 16) [74]. The researchers were guided by the premise that copper ions induce inhibition of PGE_2_ synthase, which leads to a shift in prostaglandin synthesis from pro-inflammatory PGE_2_ to the anti-inflammatory PGF series. In the carrageenan-induced paw edema model, copper acetate was found to exhibit stronger anti-inflammatory activity than hydrocortisone [75]. Furthermore, as reported by Sorenson, copper salts of various anti-inflammatory drugs were more effective in reducing inflammation than the drugs themselves [75]. Based on these findings, Sherif’s team decided to synthesize a copper complex of sudoxicam. Sudoxicam was chosen because it is a potent and preferential COX-2 inhibitor.

The team first synthesized sudoxicam and its copper complex, then evaluated their anti-inflammatory activity against COX-2 using in vitro macrophage-like P388D1 cells. Reference compounds included piroxicam, meloxicam, and copper acetate. The P388D1 cell line was selected due to its ability to induce COX-2 expression and produce PGE_2_ upon stimulation with bacterial lipopolysaccharide (LPS). The study was conducted using three different doses of the compounds (1, 10, and 100 µg), and at all doses, sudoxicam demonstrated a comparable level of PGE_2_ inhibition to meloxicam and significantly stronger inhibition than piroxicam or copper acetate.

Joint inflammation and pain tolerance as well as oxidative stress and rheumatoid factor (RF) were also assessed in rats using the thiobarbituric acid (TBA) test. Results of these studies are presented in Table 12. These experiments showed that both sudoxicam and its copper complex increased pain tolerance. Sudoxicam exhibited weaker analgesic activity, while its copper complex showed stronger effects compared to the reference drug piroxicam. Both compounds, sudoxicam and its copper complex, also demonstrated significant anti-inflammatory activity by reducing the level of RF, and this effect was stronger than that of piroxicam, which is considered a very potent anti-inflammatory drug. Moreover, the studies revealed that both compounds exhibited stronger antioxidant properties compared to piroxicam. Sherif’s comprehensive findings indicate that incorporating copper ions into the 1,2-benzothiazine structure enhances anti-inflammatory, analgesic, and even antioxidant properties, emphasizing the importance of further research into these types of metal complexes.

**Figure 17:**
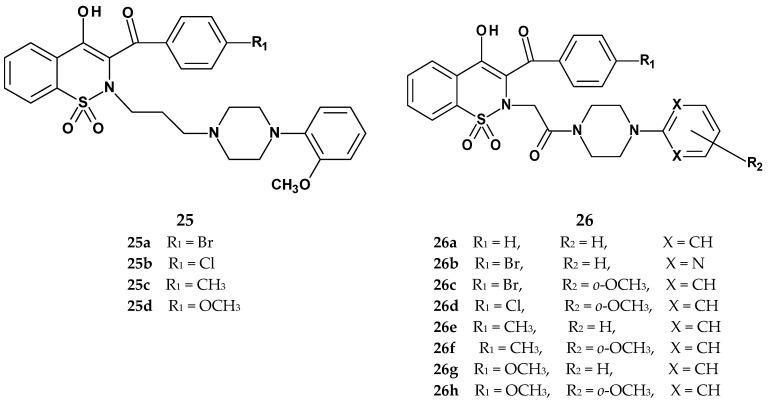
Structures of compounds **25** and **26** obtained by Szczęśniak-Sięga.

In 2021, Szczęśniak-Sięga et al., continuing their 2019 research, published the synthesis and evaluation of two new series of arylpiperazine derivatives of 1,2-benzothiazine 1,1-dioxide with general structures **25** and **26**, differing in the type of linker between the thiazine and piperazine nitrogen atoms (Figure 17) [76]. Series **25** contained a three-carbon aliphatic linker, while series **26** featured a two-carbon linker with a carbonyl group. Based on their previous work, in which they obtained 1,2-benzothiazine derivatives with anti-inflammatory and analgesic properties devoid of ulcerogenic effects, the authors aimed to further modify the lead structure and investigate the mechanism of anti-inflammatory action. They decided to synthesize new derivatives bearing an *ortho*-methoxyphenyl substituent, based on prior findings showing that compounds with this group were completely non-toxic in a mouse model [44]. The previous compounds had demonstrated significantly greater potency (2–5 times) than the reference drug piroxicam in the formalin test. However, since the earlier testing was conducted in vivo, it was not possible to determine the exact mechanism of action. The new 1,2-benzothiazine derivatives were therefore tested for COX-2 inhibition, as a pro-inflammatory target, and for COX-1, to assess selectivity.

Results showed that all tested compounds from both series exhibited COX-1 inhibitory activity, with compounds from series **26** being significantly more potent (Table 13). Compounds **26e**, **26f**, **26g**, and **26h** demonstrated even stronger COX-1 inhibition than meloxicam. In contrast, only compounds from series **26** showed activity against COX-2, which the authors attributed to the significant role of the acetyl linker between the thiazine and piperazine nitrogen atoms. The compound **26e** showed the strongest activity against COX-2, although its potency was still lower than that of meloxicam. The highest selectivity toward COX-2 was observed for compound **26b**, which is likely related to the presence of a pyrimidine ring in its structure.

The new 1,2-benzothiazine 1,1-dioxide derivatives were also tested for toxicity on normal human dermal fibroblasts (NHDF) and were found to be completely non-toxic. Additionally, studies on the interaction of these new compounds with model biological membranes demonstrated that they penetrate the phospholipid bilayer, suggesting good biological availability. Molecular docking studies to both cyclooxygenase isoforms confirmed their ability to bind to the enzyme.

**Figure 18:**
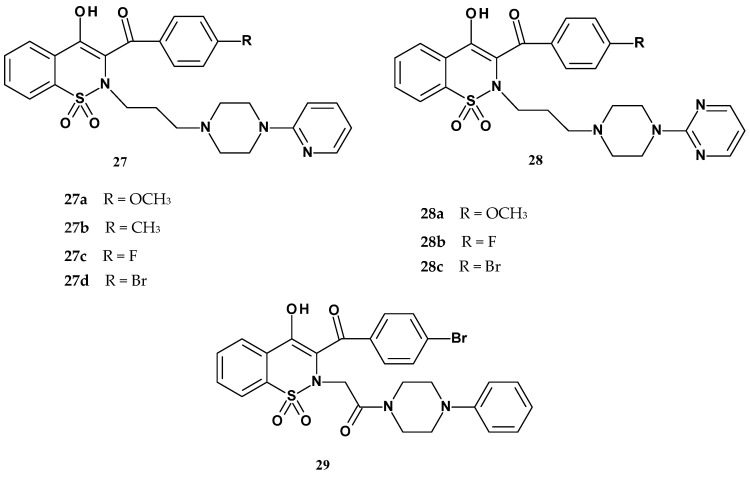
Structures of compounds **27**, **28**, and **29** obtained by Szczęśniak-Sięga.

In 2024, the Szczęśniak-Sięga team, continuing their research on 1,2-benzothiazine derivatives, published the synthesis of further new derivatives based on the structure of the most active compound **26b**, which contained a pyrimidine ring and a bromine atom [77]. Guided by this premise, the authors decided to investigate the significance of the pyrimidine ring for the activity of these derivatives and whether its replacement with a pyridine ring would affect the activity, combined with various electron-donating and electron-withdrawing substituents (Figure 18).

To compare the influence of the pyridine and pyrimidine rings on anti-inflammatory activity, one compound with a benzene ring (compound **29**) was also included in the studies. All obtained compounds were tested for their ability to inhibit both cyclooxygenase isoforms (COX-1 and COX-2) to determine the selectivity of the compounds towards these isoenzymes. A colorimetric assay was used for this purpose, with meloxicam as the reference drug. Additionally, the cytotoxicity of the tested compounds was assessed using the MTT assay. Results of the studies are presented in Table 14.

Results of the colorimetric assay showed varied effectiveness in inhibiting both COX-1 and COX-2 isoforms. However, a significantly higher affinity of the tested compounds for the COX-2 isoenzyme was observed compared to COX-1. Moreover, these compounds (except for **27b** and **28c**) exhibited stronger activity than meloxicam, which was used as the reference drug. The studies showed that compounds **27a** and **29** exhibited the most favorable COX-2 to COX-1 inhibition ratio, suggesting that the removal of the nitrogen atom from the aromatic ring may promote increased selectivity toward COX-2 by limiting interactions with COX-1. Additionally, the introduction of a halogen atom (e.g., bromine) may also positively influence affinity for COX-2. In the MTT assay, compounds **27a**, **27b**, **27c**, **27d**, **28b**, and **29** showed lower toxicity than meloxicam, with compound **27d** exhibiting the lowest toxicity among all tested.

Due to their favorable COX-2/COX-1 ratio, compounds **27a** and **29** were further studied on NHDF cell lines treated with LPS to induce pro-inflammatory cytokines. Both tested compounds significantly reduced the levels of pro-inflammatory cytokines and increased cell viability compared to the control, with compound **27a** demonstrating a somewhat stronger protective effect than **29**. Subsequently, their antioxidant properties were assessed using DPPH and ABTS assays, confirming the ability of the compounds to scavenge free radicals. The results of all studies on the new arylpiperazine derivatives of 1,2-benzothiazine obtained by Szczęśniak-Sięga indicate that they represent a promising group of compounds with anti-inflammatory activity.

The Szczęśniak-Sięga team, continuing their work on 1,2-benzothiazine 1,1-dioxide derivatives, also published the synthesis and studies of tricyclic 1,2-thiazine derivatives [78]. The authors decided to obtain tricyclic 1,2-thiazine 1,1-dioxide derivatives as potential COX-2 inhibitors, based on the patent review of COX-2 inhibitors by Rabbani. This review discusses the structures of novel COX-2 inhibitors synthesized in recent years [79]. It turns out that the new COX-2 inhibitors differ from classic NSAIDs, they have a multi-ring structure consisting of 3-, 4-, or 5-membered rings. Based on this premise and the team’s previous research, they decided to expand the bicyclic structure of 1,2-benzothiazine by adding an additional ring to look for second-generation COX-2 inhibitors.

**Figure 19:**
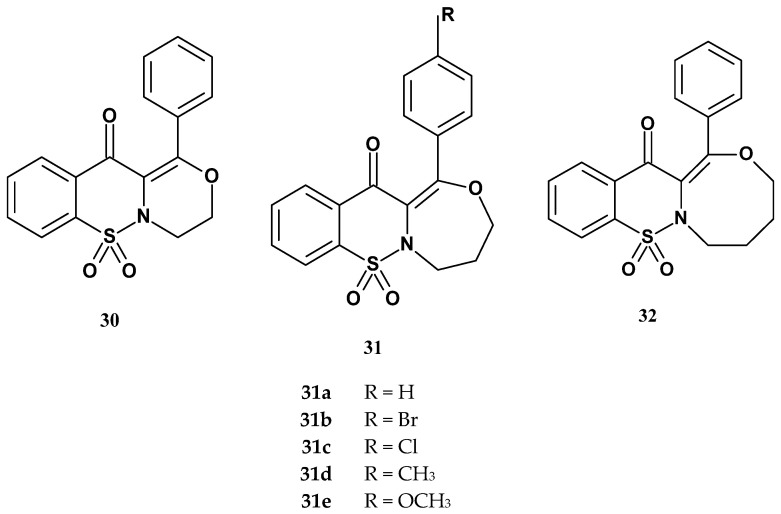
Structures of compounds **30**, **31**, and **32** obtained by Szczęśniak-Sięga.

Three series of tricyclic compounds were obtained, series **30**, **31**, and **32** (Figure 19). Series **30** contained one tricyclic 1,2-thiazine derivative with an additional six-membered oxazine ring. Series **31** included five new 1,2-thiazine derivatives with a third seven-membered oxazepine ring attached. In series **32**, there was a tricyclic compound consisting of 1,2-thiazine and an eight-membered oxazocine ring.

After obtaining seven new tricyclic 1,2-thiazine 1,1-dioxide derivatives, the authors tested their activity against COX-1 and COX-2 using a colorimetric assay. To compare the influence of the third ring on activity, bicyclic derivatives (**Xa**, **Xb**, and **Xc**) were also included in the study. The **X** compounds were 1,2-benzothiazine derivatives substituted at position 3 of the thiazine ring with benzoyl (**Xa**), 4-chlorobenzoyl (**Xb**), and 4-methylbenzoyl (**Xc**) groups, respectively. Meloxicam was used as the reference compound. Results are presented in Table 15. Cyclooxygenase inhibition studies showed that both bicyclic and tricyclic derivatives inhibited COX-1 and COX-2 activity. The vast majority of the tested compounds exhibited preferential inhibition of COX-2, with compounds **Xa**, **Xb**, **Xc**, **30**, **31e**, and **32** demonstrating stronger activity against this enzyme than meloxicam. It was therefore found that the presence of a third ring did not increase the compounds’ affinity for COX-2, as both bicyclic (**Xa**–**c**) and tricyclic (**30**, **31e**, **32**) compounds showed similarly high COX-2 inhibitory potency.

Among the tricyclic derivatives, the authors noted a certain correlation between the size of the third ring and COX-2 inhibitory properties. Derivatives containing the additional seven-membered oxazepine ring **31a**–**d** (except **31e**) exhibited weaker activity compared to compounds with the six-membered oxazine ring (**30**) or the eight-membered oxazocine ring (**32**). Interestingly, compound **31e**, which contains an oxazepine ring and a methoxy substituent in its structure, showed the highest selectivity toward COX-2, suggesting that the type of substituent may also play a significant role in this activity. As in previous studies, the authors also conducted toxicity tests of the new compounds on the NHDF cell line, assessed their interactions with model biological membranes, and performed molecular docking studies to both COX isoforms. These studies demonstrated the absence of toxicity, good membrane penetration ability, and interactions with the binding pockets of both COX-1 and COX-2.

Continuing the research on the tricyclic 1,2-thiazine 1,1-dioxide derivatives obtained by Szczęśniak-Sięga, in 2022 the Wiatrak team published a study on the effects of these compounds on neuroinflammation processes, using SH-SY5Y and THP-1 cell lines differentiated into a neuron-like phenotype [80]. The cells were incubated with bacterial lipopolysaccharide (LPS) or microglial cell supernatant to induce inflammation. The effect on cell viability was assessed in the MTT assay. The levels of free oxygen radicals (ROS) and nitric oxide (NO) were assessed by the DCF-DA and Griess assays, respectively. Since the level of ROS and NO can cause damage to the DNA strand, the FHA (Fast Halo Assay) assay was also performed, which allows assessing the DNA damage level. Computational studies on the blood–brain barrier (BBB) permeation, prediction of ADMET properties and molecular docking with TLR4/MD-2 complex were also performed. Toll-like receptors (TLRs) play an important role in inflammatory, autoimmune, and neurodegenerative disorders, including Alzheimer’s disease.

The obtained results of all tests performed for newly synthesized eight derivatives at a concentration of 10 μM after initial incubation with 50 μg/mL LPS were subjected to multiple-criteria decision analysis (MCDA). The MCDA results showed that compounds **30** and **31d** had the strongest beneficial neuroregenerative effect on SH-SY5Y cells, with 87.0% and 86.7% values, respectively. In addition, the compound **31e** showed similar strong activity to them (81.8%). Compound **30** contains a six-membered oxazine ring, while compounds **31d** and **31e** contain a seven-membered oxazepine ring substituted with a methyl or methoxy group, respectively (Figure 19). These results suggest that smaller molecular size favors the reduction in neuroinflammation in the brain, as the least active derivative was compound **32** containing an eight-membered oxazocine ring. Moreover, the studies showed that the new derivatives should cross BBB, which is crucial for reducing inflammation in the brain. It is worth noting that all tested compounds inhibited total COX activity and, in most cases, also COX-2 activity in LPS-stimulated SH-SY5Y cells, consistent with the findings of Szczęśniak-Sięga’s team.

### 3.2. New Mechanisms of Anti-Inflammatory Action

The classic mechanism of action of nonsteroidal anti-inflammatory drugs (NSAIDs) is primarily based on the inhibition of cyclooxygenase enzymes (COX-1 and COX-2). Despite the high effectiveness of drugs acting via this pathway, their long-term use is associated with serious adverse effects, particularly within the gastrointestinal and cardiovascular systems [81]. For this reason, an increasing number of studies are focusing on innovative anti-inflammatory mechanisms that carry a lower risk of complications. Alternative therapeutic targets include, among others: inhibition of microsomal prostaglandin E_2_ synthase-1 (mPGES-1), inhibition of mitogen-activated protein kinases (MAPKs), and suppression of pro-inflammatory cytokines such as TNF-α, IL-6, IL-8, IL-1β, and MCP-1.

#### 3.2.1. Inhibition of Microsomal Prostaglandin E_2_ Synthase-1 (mPGES-1)

**Scheme 2:**
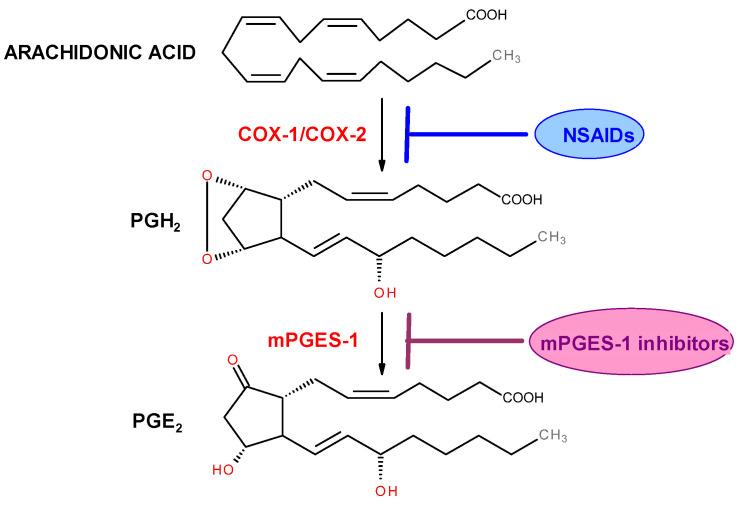
General mechanism of action of COX-1/COX-2 and mPGES-1.

A new approach in the therapy of inflammation-based diseases is the inhibition of the selectively induced microsomal prostaglandin E_2_ synthase-1 (mPGES-1). This enzyme, similarly to COX, participates in the arachidonic acid metabolic pathway, but acts at a later stage, catalyzing the biosynthesis of PGE_2_ from PGH_2_ (Scheme 2). Microsomal prostaglandin E_2_ synthase (mPGES, EC 5.3.99.3) belongs to the class of enzymes known as isomerases. It is membrane-associated and involved in the metabolism of eicosanoids and glutathione. mPGES exists in three isoforms: mPGES-1, mPGES-2, and cPGES [82].

mPGES-1 isoform is present at low levels in the prostate, testes, placenta, mammary gland, and bladder. A high expression level of this isoenzyme is observed in two cancer cell lines: cervical epithelial cells and fibroblasts [83]. mPGES-1 is the terminal enzyme in PGE_2_ biosynthesis and requires the presence of glutathione (GSH) as an essential cofactor for its enzymatic activity [82].

mPGES-2 isoform is found in the brain, skeletal muscles, kidneys, and liver. This isoform couples with COX-1 or COX-2, leading to PGE_2_ synthesis [83].

cPGES isoform is a cytosolic protein involved in PGE_2_ production in response to stimuli such as Ca^2+^ ions [84]. This enzyme is constitutively expressed and is not affected by pro-inflammatory stimuli [85].

All three isoforms, mPGES-1, mPGES-2, and cPGES, catalyze the conversion of PGH_2_ to PGE_2_. However, mPGES-1 is activated in response to inflammatory stimuli and is the main factor responsible for PGE_2_ production during inflammation. For this reason, selective inhibitors of mPGES-1 are expected to suppress inflammation-induced PGE_2_ biosynthesis while not affecting the production of constitutive PGE_2_ and other prostanoids (PGI_2_, TX), which should translate into fewer adverse drug effects [86]. Moreover, recent studies indicate the involvement of mPGES-1 in carcinogenesis. It has been shown that this enzyme is overexpressed in brain, breast, kidney, lung, and endometrial cancers, as well as in gastrointestinal cancers such as esophageal, stomach, and colorectal cancer [87]. Therefore, inhibiting mPGES-1 activity may provide not only anti-inflammatory but also anticancer effects.

**Figure 20:**
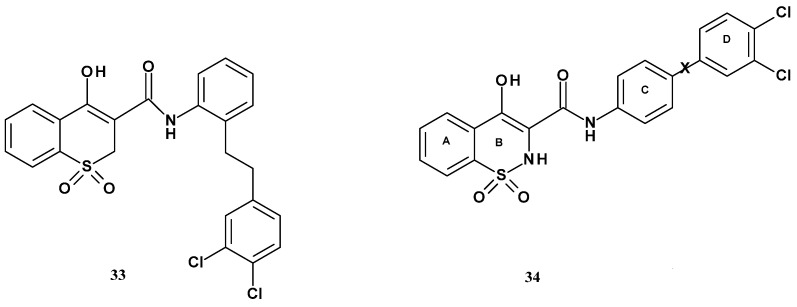
The structure of the 1,1-dioxide benzothiopyran derivative (**33**) and 1,2-benzothiazine derivatives (**34**) obtained by Wang.

In 2010, the Wang team published the synthesis and evaluation of new 1,2-benzothiazine 1,1-dioxide derivatives targeting mPGES-1 inhibition [88]. Wang based the work on results from high-throughput screening conducted by Pfizer, which showed that 1,1-dioxide benzothiopyran derivatives exhibited mPGES-1 inhibitory properties. Moreover, these compounds demonstrated greater selectivity for mPGES-1 over COX-2 in assays using IL-1-stimulated fetal fibroblast cells. Inspired by these compounds, the Wang team decided to replace the benzothiopyran ring with a 1,2-benzothiazine ring to obtain compounds with increased selectivity and inhibitory potency toward mPGES-1 (Figure 20).

The Wang team planned various modifications of the substituent at position 3 of the thiazine ring. The first modification involved introducing different linkers between rings C and D. The resulting derivatives were then tested for mPGES-1 inhibition. Results of these studies are presented in Table 16.

The modification of the model compound **33** began with the removal of the ethyl linker between rings C and D, as well as the removal of the chlorine atom at position 3. The resulting compound **35a** exhibited three times lower mPGES-1 inhibitory activity compared to the unmodified compound. Based on these data, further analogs were designed with a linker X introduced between rings C and D in the form of an oxygen atom, an amino group, or a carbonyl group (**35b**–**e**) to investigate the effect of this linker on mPGES-1 inhibition. Compound **35b** containing an oxygen atom (X = O) showed a twofold decrease in inhibitory activity compared to **35a**. Compound **35c** containing an amino group (X = NH) showed a twofold increase in inhibitory potency against the enzyme.

Among the compounds with a carbonyl group (X = C=O), **35d** and **35e**, the *para*-substituted analog (**35d**) was the strongest mPGES-1 inhibitor in this series, whereas the *meta*-substituted analog (**35e**) was the weakest. These data suggest that the nature of the linker does not significantly affect mPGES-1 inhibition, while the *para* position of the substituent is clearly preferred over the *meta* position.

The second modification of the new 1,2-benzothiazine derivatives conducted by Wang’s team involved introducing various substituents into ring D. Results of mPGES-1 inhibition by ten compounds obtained in this series are presented in Table 17.

The studies showed that the nature and position of the substituents within ring D were important for the strength of mPGES-1 inhibition. Compound **35a** with a chlorine atom at the *para* position inhibited mPGES-1 more strongly than its counterpart with chlorine at the *meta* position (**36a**). Disubstituted analogs with halogen atoms (**36e**, **36f**, and **36g**) were stronger inhibitors of mPGES-1 than the mono-substituted analogs (**35a** and **35a**). Furthermore, the chlorine atom (in compound **36a**) and the methyl group (**36b**) proved to be more favorable substituents than hydrogen (**36d**), cyano (**36c**), or methoxy group (**36h**). Meanwhile, the effect of methyl substituent (**36i** and **36j**) depended on its position. Among the compounds in this series, the strongest mPGES-1 inhibitor was analog **36e**.

**Figure 21:**
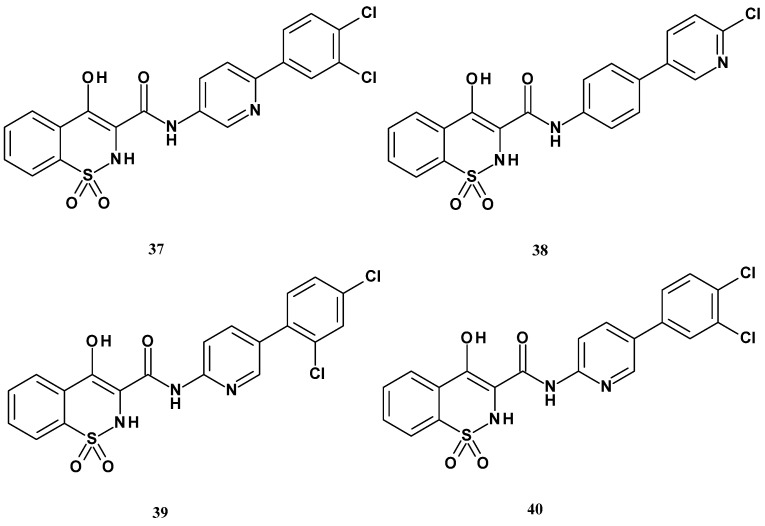
Structures of compounds obtained by Wang as a result of the third modification.

The third modification carried out by Wang’s team involved introducing pyridine in place of the benzene ring C or D. The four compounds obtained in this way are shown in Figure 21.

Results of the mPGES-1 inhibition studies for the compounds of the third series are presented in Table 18. They showed a significant decrease in activity against mPGES-1, leading to the conclusion that the biphenyl group is much more favorable for this activity.

The fourth modification carried out by the Wang team involved introducing various substituents to ring A as well as modifications within ring B, including ring opening. In this way, Wang obtained eight compounds, six of which were 1,2-benzothiazine derivatives, and two had an opened thiazine ring. Results of mPGES-1 inhibition by compounds from this series are presented in Table 19. Analogs with substituents in ring A (**41a**, **41b**, **41c**, and **41d**) did not show an increased inhibitory strength against mPGES-1 compared to the most potent compound **36e** (see Table 17). However, within these four compounds replacing the methoxy group (**41a** and **41c**) with a hydroxyl group (**41b** and **41d**) resulted in better activity, but still weaker than compound **36e**. Compound **41e**, with a methyl substituent at position 2 of the thiazine ring, was designed to improve water solubility, but this resulted in a strong decrease in activity against mPGES-1. Compound **41f** showed good water solubility but also low activity against mPGES-1.

**Figure 22:**
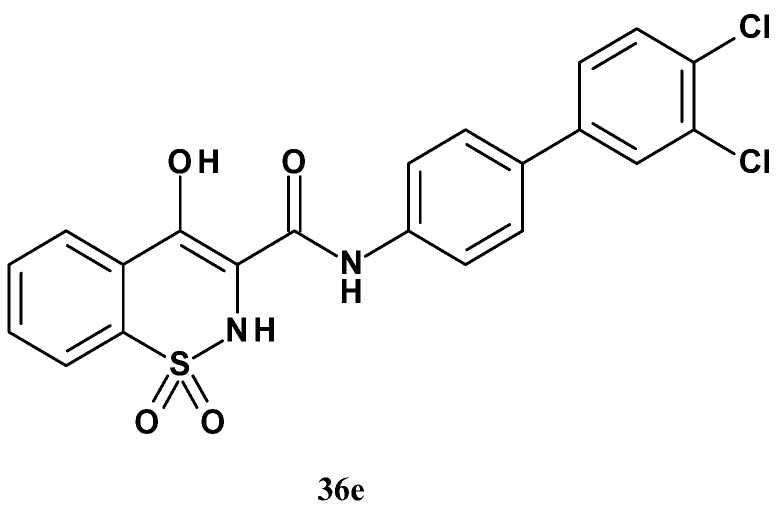
Structure of compound **36e** studied by Mbalaviele.

Continuing Wang’s research, the Mbalaviele team focused on the most active compound, **36e** (Figure 22). This team conducted in-depth studies of the activity of compound **36e** in the carrageenan test, LPS-stimulated human whole blood, unstimulated human whole blood, and a modified human blood assay [89]. This compound proved to be a strong inhibitor of recombinant human (rh) mPGES-1, without affecting rhCOX-1 and rhCOX-2. To study the expression of mPGES-1 in vivo during inflammation, carrageenan was administered at various time intervals into air pouches created in rats by subcutaneous injection of sterile air. Mbalaviele evaluated the efficacy of compound **36e** in this model by conducting several trials assessing its effect on PGE_2_ synthesis after oral or local administration into the air pouches, but these attempts were unsuccessful.

Results allowed for determining the inhibitory strength of compound **36e** on recombinant mPGES-1. It was found that the compound **36e** is a weak inhibitor of recombinant rat mPGES-1 (IC_50_ = 1080 nM) compared to recombinant human mPGES-1 (IC_50_ = 16.5 nM). In the next stage, Mbalaviele assessed the selectivity and efficacy of compound **36e** in tests on human blood. In the first test using LPS-stimulated human whole blood, compound **36e** weakly inhibited PGE_2_ synthesis and showed no effect on thromboxane B_2_ (TXB_2_) synthesis. Then, the effect of this inhibitor was examined in a test with unstimulated human whole blood, where prostaglandin synthesis depends on exogenously added arachidonic acid and is mainly driven by COX-1. In this test, compound **36e** was completely ineffective.

Finally, the efficacy of the compound was evaluated in a modified human blood assay. To create a condition with rapid PGE_2_ synthesis, the test was modified by adding cells derived from squamous cell carcinoma of the head and neck, which were then exposed to arachidonic acid for 10 min. It turned out that the addition of cancer cells to the blood restored the ability of compound **36e** to inhibit PGE_2_ synthesis. Based on these data, Mbalaviele concluded that compound **36e** selectively inhibited PGE_2_ synthesis via mPGES-1 while sparing TXB_2_ synthesis.

#### 3.2.2. Inhibition of Pro-Inflammatory Cytokines

Cytokines are small proteins produced by various types of immune system cells. Their main role is to transmit signals in an autocrine or paracrine manner, modulating the local activity of immune cells, including survival, growth, and differentiation [90]. There are both pro-inflammatory and anti-inflammatory cytokines. The former are produced primarily by monocytes and macrophages and are involved in the regulation of inflammatory responses, for example, by activating leukocytes, increasing the permeability of blood vessels, and inducing fever. These include interleukin 1β (IL-1β), interleukin-6 (IL-6), interleukin-8 (IL-8), tumor necrosis factor alpha (TNF-α), and monocyte chemoattractant protein 1 (MCP-1) [91]. These cytokines play a significant role in the pathogenesis of many inflammatory, autoimmune, and neoplastic diseases; their chronic release is associated with, among others, impaired neurogenesis, atherosclerosis, rheumatoid arthritis, Alzheimer’s disease, and even cancer [92,93,94,95]. This suggested that the search for new chemical compounds targeting the inhibition of pro-inflammatory cytokines represents a promising strategy in the treatment of many diseases in which inflammation plays a key role.

**Figure 23:**
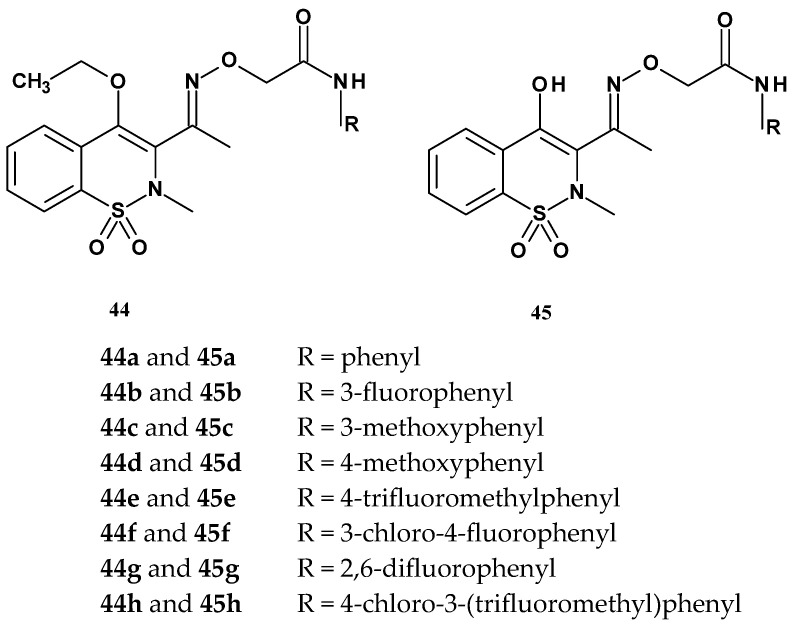
Structures of ether derivatives of 1,2-benzothiazine obtained by Gannarapu.

In 2014, the Gannarapu team published the synthesis and studies on the anti-inflammatory activity and the ability to inhibit monocyte-to-macrophage differentiation of new oxime-structured 1,2-benzothiazine 1,1-dioxide ether derivatives. The authors selected the 1,2-benzothiazine scaffold substituted at position 3 with an oxime group due to its favorable biological significance [93]. Gannarapu et al. obtained two series (**44** and **45**) of *N*-arylacetamide ether derivatives of 1,2-benzothiazine, with general structures presented in Figure 23. The first series of compounds (**44a**–**h**) contained an ethoxy group at position 4 of the thiazine ring, while the second series (**45a**–**h**) had a hydroxyl group. The new derivatives differed in both series by the type of substituent attached to the nitrogen in the amide group (R), which was a phenyl group bearing either electron-donating (OCH_3_) or electron-withdrawing (Cl, F, CF_3_) substituents.

After synthesis, all compounds were evaluated for their anti-inflammatory activity in a PMA (phorbol 12-myristate 13-acetate)-induced inflammation model. The study involved assessing the levels of pro-inflammatory cytokines such as TNF-α, IL-8, and MCP-1 using the THP-1 monocyte cell line, measured via ELISA assay. Piroxicam and pioglitazone were used as reference compounds. Results are presented in Table 20.

The study showed that most of the tested compounds did not exhibit activity against any of the cytokines. Only four compounds were active, **44e**, **45b**, **45e**, and **45h**. Compound **45b** inhibited the secretion of MCP-1 only, while compounds **44e**, **45e**, and **45h** affected two types of cytokines: **44e** inhibited IL-8 and MCP-1, and **45e** and **45h** inhibited TNF-α and MCP-1. Of particular note was the strong activity of compounds **45e** and **45h** against MCP-1, with IC_50_ values of 4.0 µM and 2.5 µM, respectively. Therefore, their effect on the activity of matrix metalloproteinases (MMP-9), induced by PMA during the transformation of monocytes into macrophages, was further investigated.

MMP-9 plays a significant role in various diseases, including cancers and atherosclerosis. A gelatin zymography assay was used to assess MMP-9 activity, and the compounds were applied at a concentration of 10 µM. This study showed that both **45e** and **45h** inhibited MMP-9 activity, with **45h** being more potent, as it inhibited MMP-9 activity by 75%. For both compounds, an additional inhibition of PMA-induced monocyte to macrophage transformation assay was performed, because transformed monocytes initiate inflammatory responses by secreting various pro-inflammatory cytokines. It was found that, pretreatment of compounds **45e** and **45h** at 10 µM concentration significantly inhibited the PMA-induced increase in cell adherence, cell size, mitochondrial number, LOX-1 and CD-36 expressions in THP-1 cells. These results indicate that the presence of a trifluoromethyl group and a chlorine atom on the phenyl ring, in combination with a hydroxyl group at position C-4, plays a key role in the observed biological activity.

**Figure 24:**
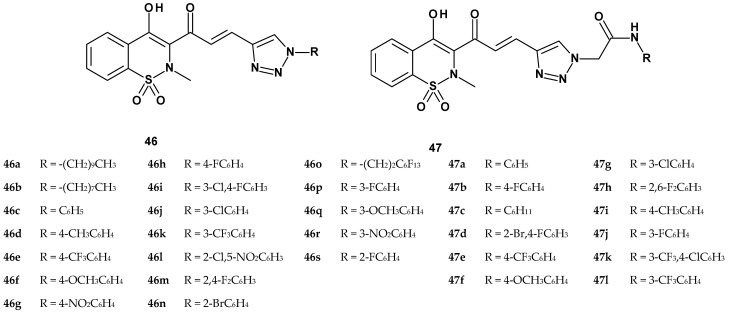
Structures of the new 1,2-benzothiazine derivatives obtained by Gannarapu.

In 2015, Gannarapu et al. published the synthesis and biological evaluation of two new series of 1,2-benzothiazine 1,1-dioxide derivatives [96]. Building on their previous research, the authors aimed to further modify the 1,2-benzothiazine scaffold to obtain even more potent inhibitors of pro-inflammatory cytokine secretion. Two new series (**46** and **47**) of propenone derivatives of 1,2-benzothiazine were synthesized, each featuring a 1,2,3-triazole ring attached at position 3 of the benzothiazine core. The general structures of these compounds are shown in Figure 24. Series **46** comprised 19 compounds, while series **47** included 12 compounds.

The compounds from series **46** differed from those in series **47** by the absence of an acetamide group at position 1 of the triazole ring. Additionally, both series contained various substituents (aliphatic and aromatic): in series **46**, these substituents were directly attached to the nitrogen at position 1 of the triazole, whereas in series **47**, they were attached to the nitrogen atom within the acetamide moiety. The compounds synthesized by the Gannarapu team were tested for their ability to inhibit the production of pro-inflammatory cytokines such as TNF-α, IL-1β, and MCP-1 in PMA-stimulated monocytes. The results were expressed as the percentage of inhibition compared to the control group treated with PMA alone (Table 21). Piroxicam and celecoxib were used as reference drugs.

The study results showed that compounds from both series were more active against IL-1β and MCP-1 cytokines than TNF-α. Compounds from series **47** demonstrated greater efficacy in inhibiting pro-inflammatory cytokines compared to series **46**, as they inhibited the secretion of all three types of pro-inflammatory cytokines much more strongly than both reference compounds. Moreover, all compounds from both series did not negatively affect cell viability. Among the compounds from series **47**, the strongest effects were shown by **47e**, **47g**, **47i**, **47j**, **47k**, and **47l**, therefore the authors decided to further evaluate their ability to inhibit PMA-induced pro-inflammatory cytokines in a dose-dependent manner (at five concentrations: 2.5, 5, 10, 15, and 20 µM). Studies showed that all compounds inhibited cytokine production in a dose-dependent manner.

The anti-inflammatory activity was further confirmed by PMA-induced COX-2 mRNA expression. All these compounds significantly inhibited the transcript levels of PMA-induced COX-2. These results suggest that promising compounds with inhibitory effects on pro-inflammatory cytokine profiles also affect COX-2 signaling at the transcription level. Next, their effect on PMA-induced monocyte-to-macrophage differentiation was examined by treating monocytes with compounds **47e**, **47g**, **47i**, **47j**, **47k**, and **47l** (5–20 μM). From the phase contrast images, it was observed that compounds **47g**, **47i**, and **47l** at 15 μM concentration significantly inhibited PMA-induced cell adherence, the PMA-induced increase in cell size as reflected by the transcript levels of PMA-induced LOX-1 and CD-36 scavenger receptors (markers of monocyte differentiation). Treatment of monocytes with these three compounds greatly inhibited PMA-induced lysosomal activity, thereby indicating that these compounds inhibit PMA-mediated monocyte-to-macrophage differentiation.

As compounds **47g**, **47i**, and **47l** showed an excellent inhibitory effect on the differentiation of monocytes, they further examined the beneficial effects of these compounds by measuring PMA-induced MMP-1 and MMP-9 transcript levels using RT-PCR. It was found that these three compounds significantly inhibited the transcript levels of both MMP-1 and MMP-9. Analyzing the structure of the most active compounds, it can be noted that the presence of the acetamide group on the triazole ring is very important, and the presence of substituents: 3-chloro (**47g**), 4-methyl (**47i**), and 3-trifluoromethyl (**47l**) additionally enhances the inhibition of the inflammatory response at many stages. Finally, authors concluded that compounds **47g**, **47i**, and **47l** may have beneficial effects in mitigating inflammation-associated disorders upon further validation.

#### 3.2.3. Inhibition of MAP Kinases

MAP kinases (MAPK) are a group of serine/threonine protein kinases that play a key role in regulating the cellular response to external signals (mitogens). Through phosphorylation, they regulate the activity of many proteins, thereby influencing critical cellular processes such as gene expression, cell division and differentiation, as well as cell survival or death [97]. The four main groups of MAP kinases include: ERK (extracellular signal-regulated kinases), JNK (c-Jun N-terminal kinases), p38 protein isoforms, and ERK5 (extracellular signal-regulated kinase 5) [98].

The p38 isoforms play a particularly important role in regulating the production of pro-inflammatory cytokines such as TNF-α, IL-1β, and IL-6, which are key mediators in autoimmune and inflammatory diseases such as rheumatoid arthritis (RA), systemic lupus erythematosus (SLE), chronic obstructive pulmonary disease (COPD), and type 1 diabetes [99,100,101]. Recent studies also suggest that p38 kinases may play a significant role in the development of cancers [102,103] and neurodegenerative diseases such as Alzheimer’s disease [104] and Parkinson’s disease [105].

**Figure 25:**
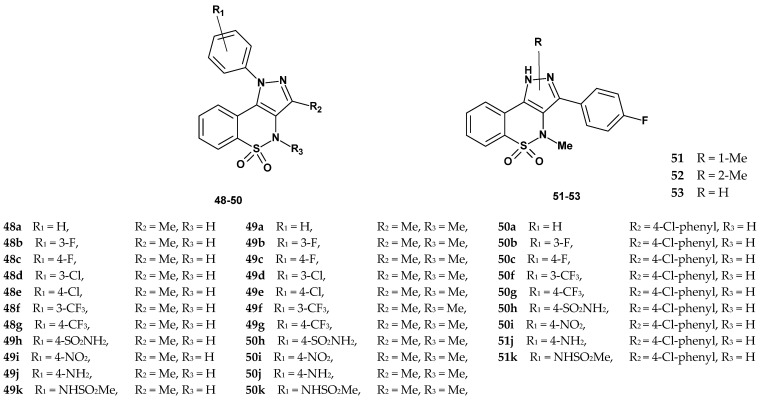
Structures of the new pyrazolobenzothiazine derivatives obtained by Sabatini.

In 2015, Sabatini et al. published the synthesis and evaluation of new tricyclic 1,2-benzothiazine 1,1-dioxide derivatives as MAP kinase inhibitors [106]. The authors designed a hybrid structure combining a bicyclic 1,2-benzothiazine scaffold with a pyrazole ring (Figure 25), inspired by celecoxib, a selective COX-2 inhibitor. The new compounds were initially tested for COX-2 inhibition but turned out to be inactive. However, their structure also resembled **SB203580**, which is a specific inhibitor of p38α and p38β that suppresses downstream activation of MAPKAP kinase-2 and heat shock protein 27 [107]. Therefore, the authors decided to investigate the new compounds for their potential to inhibit p38 MAPK.

Sabatini et al. obtained six series of new 1,2-benzothiazine derivatives (**48**, **49**, **50**, **51**, **52**, and **53**), with series **51**, **52**, and **53** consisting of single compounds. In the compounds of series **48**–**50**, the phenyl ring was attached to the nitrogen atom (N-1) of the pyrazole, whereas in series **51**–**53**, it was located at position 3 of the pyrazole ring. Moreover, in series **51**–**53**, the phenyl ring contained only a fluorine atom in the *para* position, while in series **48**–**50**, various substituents (Cl, F, CF_3_, NH_2_, SO_2_NH_2_, NHSO_2_Me) were present at different positions. Series **48** and **49** each contained 11 compounds (**48a**–**k** and **49a**–**k**), differing in the presence or absence of a methyl group on the thiazine nitrogen atom. Series **50** consisted of 9 compounds (**50a**–**c**, **50f**–**k**) and was characterized by the presence of a *para*-chlorophenyl substituent at position 3 of the pyrazole ring.

All synthesized compounds were evaluated for their ability to inhibit p38α MAPK activity and to suppress TNF-α secretion in human whole blood (HWB) stimulated with LPS. Results of the enzymatic tests confirmed that most of the new pyrazolobenzothiazine derivatives inhibit p38 MAPK activity (Table 22). Compounds that reduced the activity of p38α kinase by more than 65% at a concentration of 10 µM were considered active. This group included derivatives: **48d**, **48f**, **48i**, **48k**, **50a**–**c**, **50f**, **50h**, **50i**, **50k**, as well as compounds **51**, **52**, and **53**, which were then tested at various concentrations to determine their IC_50_ values. The reference compound was **SB203580**, with an IC_50_ = 0.05 µM.

Compounds **48d**, **48f**, **48i**, **48k**, **50a**–**c**, **50f**, **50h**, **50i**, **50k**, **51**, **52**, and **53** demonstrated effectiveness as inhibitors, with IC_50_ values ranging from 0.43 to 7.73 µM. The study results showed that the compounds of series **49** turned out to be very weak inhibitors. Since they differed from the compounds of series **48** only by the presence of a methyl group at the thiazine nitrogen atom, it can be concluded that this substituent is unfavorable for the activity.

In contrast, almost the entire series **50** (except for **50g** and **50j**) containing a *para*-chlorophenyl substituent at the R_2_ position proved to be very active, indicating that this group in this position is crucial. However, the compounds of series **51**, **52**, and **53** exhibited the highest activity, suggesting that substitution with an aromatic ring at the 3-position of the pyrazole is significantly more favorable than substitution at the N-1 position. It can also be inferred that the presence of *meta*-trifluoromethyl, *para*-nitro, and *para*-methylsulfonamide groups in the phenyl ring enhances this activity.

Two of the most active compounds, **53** and **48d**, were selected for further in-depth studies. For these two compounds, inhibition assays were carried out against other kinases (CDC2, CDK5, ERK1, JAK2, JAK3, LCK, SRC, p38-β, p38-δ, p38-γ, PDGFRβ, GSK3-β, PKAC-α) to determine their selectivity profile. It was found that both compounds are selective toward p38 protein, but compound **53** was stronger than **48d** (with IC_50_ values of 1.7 µM and 16 µM, respectively). Additionally, molecular docking studies with p38 MAPK were performed to determine their binding mode. Overall, the authors conclude that derivative **53**, with a reasonable kinase selectivity profile, low molecular weight, and ligand efficiency values, make it an attractive starting point for chemistry efforts aimed at further potency optimization.

## 4. Summary

The conducted literature review confirmed that compounds containing the 1,2-benzothiazine 1,1-dioxide scaffold exhibit anti-inflammatory activity in both in vitro and in vivo studies. In vivo studies using various assays have shown that these compounds have anti-inflammatory activity. While in vitro studies provided insights into the mechanisms responsible for this activity. It has been shown that new 1,2-benzothiazine 1,1-dioxide derivatives can act through the classical mechanism, by inhibiting cyclooxygenase (COX-1 and COX-2), as well as through more modern mechanisms, which include inhibition of microsomal prostaglandin E_2_ synthase-1 (mPGES-1) or 11β-hydroxysteroid dehydrogenase type 1 (11β-HSD1), suppression of pro-inflammatory cytokine release (e.g., TNF-α, IL-1β, IL-8), and modulation of protein kinase activity, including members of the MAP kinase family. Due to the different types of research, it is difficult to identify a single leading structure. Although within each of the discussed mechanisms of action, a compound with promising properties and potential for further in-depth research can be identified. Some of the newly synthesized 1,2-benzothiazine 1,1-dioxide derivatives demonstrated stronger anti-inflammatory effects than standard nonsteroidal anti-inflammatory drugs (NSAIDs), making them particularly promising candidates for use in modern therapies for inflammation-related diseases.

## Figures and Tables

**Table 1:** Inhibitory effects of compounds **1a**–**1f** on antigen-induced histamine release.

Compound	Inhibition (%)
**1a**	47.7
**1b**	25.2
**1c**	21.7
**1d**	72.3
**1e**	94.2
**1f**	102.7
tranilast	42.2
piroxicam	92.5

**Table 2:** Results of the carrageenan-induced edema and writhing tests conducted by Kwon’s team.

Compound	Antiinflammatory ^a^	Analgesic ^b^	Compound	Antiinflammatory ^a^	Analgesic ^b^
**2a**	IN	IN	**2m**	MO	MK
**2b**	IN	SL	**2n**	MO	IN
**2c**	IN	SL	**2o**	MO	SL
**2d**	IN	MO	**2p**	IN	IN
**2e**	IN	SL	**2q**	IN	SL
**2f**	IN	SL	**2r**	IN	MO
**2g**	IN	MO	**2s**	MO	MO
**2h**	MK	MK	**2t**	IN	SL
**2i**	MK	MK	**2u**	IN	IN
**2j**	MK	MK	indomethacin	MK	MO
**2k**	MK	MK	aspirin	-	MO
**2l**	MK	MO			

^a^ % inhibition of edema: MK = marked activity (>45); MO = moderate activity (35~44); IN = insignificant activity (<20); ^b^ % inhibition of writhing: MK (>60); MO (50~59); SL (40~49); IN (<39); significant *p* < 0.01.

**Table 3:** Results of the studies of compounds **3** obtained by Lee.

Treatment	Carrageenan Test (Increase Percent of Paw Volume)				Randall–Selitto Assay ED_50_ (mg/kg)	
	1 h	2 h	3 h	4 h	30 min	60 min
**3a**	64.1 ± 9.5	67.0 ± 7.8	82.8 ± 6.8	92.8 ± 10.6	-	-
**3b**	71.6 ± 2.4	77.5 ± 1.1	80.1 ± 1.9	87.3 ± 2.4	79.9	215.9
**3c**	69.0 ± 5.8	70.4 ± 5.8	73.0 ± 5.9	82.0 ± 5.8	34.7	944.5
control	56.9 ± 6.1	57.8 ± 6.1	75.3 ± 10.6	79.2 ± 7.5	-	-
piroxicam	39.2 ± 4.0	40.1 ± 4.9	39.0 ± 3.7	48.1 ± 6.6	5.2	37.5
ibuprofen	-	-	-	-	120.1	166.6

All data represent the mean ± S.E.M.

**Table 4:** Results of the carrageenan test for compounds **4**–**6** obtained by Sharma.

Compound	Inhibition (%)			
	p.o.		i.p.	
	2 h	3.5 h	2 h	3.5 h
**4a**	19	-	34	35
**4b**	17	5	61	47
**4c**	-	-	30	37
**4d**	-	5	61	40
**4e**	7	22	65	64
**4f**	36	22	53	34
**5a**	32	19	66	46
**5b**	41	16	53	40
**5c**	34	22	38	29
**6**	30	12	53	59
ibuprofen	62	65	70	74

**Table 5:** Results of the writhing and carrageenan assay for selected compounds obtained by Maso.

Compound	R_1_	R_2_	Writhing Assay	Carrageenan Assay
			ID_50_	Inhibition (%)
**7b**	CH_2_N(CH_2_CH_3_)_2_	phenyl	20.20	76.47
**7c**	CO_2_CH_2_CH_3_	phenyl	6.07	81.51
**7d**	CH_2_CO_2_CH_2_CH_3_	phenyl	7.24	82.36
**7e**	CN	phenyl	17.50	77.31
**7j**	CH_2_N(CH_2_CH_3_)_2_	isopropyl	>50	54.54
**7k**	CH_2_N(CH_3_)_2_	isopropyl	>50	53.78
piroxicam	H	pyridyl	0.76	85.71

**Table 6:** Results of the carrageenan test for compounds from series **8** and **9** obtained by Kacem.

Compound	Inhibition (%) after 3 h	ED_50_
**8a**	27.8	29.8
**8b**	47.0	9.2
**9a**	32.4	23.4
**9b**	54.5	4.7
control	-	-
piroxicam	67.3	4.4

**Table 7:** Results of in vivo tests and COX inhibition for compounds synthesized by Miranda.

Compound	Inhibition (%)					
	The Writhing Test	The Formalin Test Phase 2	The Zymosan Test	Carrageenan Test	COX-1	COX-2
**10a**	76.1	11.1	43.7	57.4	-	-
**10b**	58.6	38.4	73.9	22.5	N/A	N/A
**10c**	75.1	9.1	56.6	62.2	-	-
**10d**	57.9	16.8	72.1	42.6	-	-
**10e**	90.4	32.5	37.1	74.2	-	-
**10f**	62.7	60.1	78.7	73.2	N/A	N/A
**10g**	84.0	54.2	81.3	77.5	-	-
**10h**	82.7	27.5	82.6	81.8	-	-
piroxicam	95.4	53.9	57.4	25.8	-	-

N/A = not active.

**Table 8:** Results of the formalin test for compounds obtained by Szczęśniak-Sięga.

Compound	ED_50_ (mg/kg) Early Phase	ED_50_ (mg/kg) Late Phase	Compound	ED_50_ (mg/kg) Early Phase	ED_50_ (mg/kg) Late Phase
**11a**	11.59	6.18	**12a**	13.95	8.85
**11b**	27.02	7.14	**12b**	-	7.93
**11c**	-	9.03	**12c**	22.78	7.22
**11d**	-	4.01	**12d**	12.83	6.24
piroxicam	-	18.85	**12e**	11.92	8.25

**Table 9:** Results of COX-1 and COX-2 inhibition studies by series I meloxicam analogs.

Compound	R_1_	R_2_	Ar	COX-1 (%)	COX-2 (%)
**13a**	H	CH_3_	2-thiazole	51	35
**13b**	H	CH_3_	5-ethyl-2-thiazole	30	1
**13c**	H	CH_3_	5-phenyl-2-thiazole	97	19
**13d**	H	CH_3_	5-methyl-2-thiadiazole	2	23
**13e**	H	CH_3_	4-methyl-2-oxazole	−7	−7
**13f**	H	CH_3_	5-methyl-3-isoxazole	10	9
**13g**	H	CH_3_	phenyl	26	−18
**13h**	6-OCH_3_	CH_3_	5-methyl-2-thiazole	−36	40
**13i**	6-CH_3_	CH_3_	5-methyl-2-thiazole	24	50
**13j**	6-Cl	CH_3_	5-methyl-2-thiazole	62	76
**13k**	6-F	CH_3_	5-methyl-2-thiazole	56	72
**13l**	7-OCH_3_	CH_3_	5-methyl-2-thiazole	50	70
meloxicam	H	CH_3_	5-methyl-2-thiazole	39	77
piroxicam	H	CH_3_	2-pyridine	35	62

**Table 10:** Results of COX-1 and COX-2 inhibition studies for selected meloxicam analogs from series II.

Compound	R_1_	R_2_	Ar	COX-1 (%)	COX-2 (%)
**14**	-	-	2-thiazole	69	1
**15**	-	-	2-thiazole	100	43
**16**	H	CH_2_CH_3_	2-thiazole	86	1
**17**	Cl	-	2-thiazole	100	37
**18**	-	-	2-thiazole	−24	−7
**19**	H	S	5-methyl-2-thiazole	41	77
meloxicam	-	-	-	39	77

**Table 11:** Results of COX-2 and COX-1 inhibition studies for selected meloxicam analogs from series III.

Compound	R_1_	R_2_	Ar	COX-1 (%)	COX-2 (%)
**20a**	6-Cl	methyl	2,4-dichlorophenyl	100	100
**20b**	6-Cl	methyl	2-chlorophenyl	100	100
**20c**	6-Cl	methyl	2,4-dibromophenyl	99	97
**20d**	6-Cl	benzyl	2,4-dichlorophenyl	100	92
**20e**	6-Cl	ethyl	2,4-dichlorophenyl	99	100
**20f**	6-Cl	cyclopropyl	2,4-dichlorophenyl	100	100
**21**	-	-	4-chlorophenyl	100	93
**22**	-	-	phenyl	72	13
**23**	Cl	S	4-dichlorophenyl	91	74
**24**	-	-	4-chlorophenyl	100	96
meloxicam	-	-	-	39	77

**Table 12:** Results of studies of copper complex of sudoxicam obtained by Sherif.

Group	Dose (mg/200 g)	Pain Tolerance n = 6	Proximal Joint Mobilization Tolerance n = 6	MDA (nmol/mL) n = 6	RF (IU/mL) n = 6
non-arthritic non-treated	solvent (SCMC)	1.0 ± 0.00	1.48 ± 1.18	2.74 ± 2.1	178 ± 2.1
arthritic non-treated	solvent (SCMC)	3.9 ± 0.10	3.15 ± 0.55	4.0 ± 0.39	528 ± 71.0
Cu(Ac)_2_·2H_2_O	0.22	2.5 ± 0.09	2.58 ± 0.15	2.8 ± 0.33	385 ± 21.0
piroxicam	0.36	2.1 ± 0.12	2.07 ± 0.28	2.8 ± 0.2	325 ± 10.0
sudoxicam	0.36	2.8 ± 0.14	2.58 ± 0.6	1.5 ± 0.42	258 ± 31.0
Cu complex	0.96	1.7 ± 0.09	1.67 ± 0.11	1.8 ± 0.42	190 ± 9.0

n—number of rats; SCMC—0.5% sodium carboxymethyl cellulose.

**Table 13:** Results of studies of compounds **25** and **26** obtained by Szczęśniak-Sięga.

Compound	Cyclooxygenase Inhibition Assay IC_50_ (µM)		COX Selectivity Ratio IC_50(COX-2)_/IC_50(COX-1)_
	COX-1	COX-2	
**25a**	380.5	N/A	-
**25b**	158.4	N/A	-
**25c**	192.4	N/A	-
**25d**	103.5	N/A	-
**26a**	87.6	125.2	1.43
**26b**	146.4	115.8	0.79
**26c**	101.8	N/A	-
**26d**	204.0	205.6	1.01
**26e**	82.9	94.0	1.13
**26f**	70.4	158.6	2.25
**26g**	64.1	116.7	1.82
**26h**	79.1	N/A	-
meloxicam	83.7	59.2	0.71

N/A—compound not active.

**Table 14:** Results of the studies of compounds **27**, **28**, and **29** obtained by Szczęśniak-Sięga.

Compound	Cyclooxygenase Inhibition Assay		Selectivity Ratio (COX-2/COX-1)	MTT Assay IC_50_ (µM)
	COX-1	COX-2		
	IC_50_ (µM)	IC_50_ (µM)		
**27a**	241.64 ± 4.2	13.19 ± 2.1	0.05	228.40 ± 28.1
**27b**	130.96 ± 7.5	62.63 ± 1.7	0.48	200.10 ± 20.9
**27c**	128.73 ± 9.1	18.20 ± 4.3	0.14	203.28 ± 22.9
**27d**	124.81 ± 6.7	17.80 ± 2.1	0.14	232.51 ± 11.8
**28a**	126.54 ± 3.6	26.34 ± 3.4	0.21	163.46 ± 15.7
**28b**	116.89 ± 4.3	25.55 ± 2.0	0.22	190.60 ± 12.5
**28c**	121.51 ± 3.4	59.22 ± 3.7	0.49	168.50 ± 30.4
**29**	95.88 ± 5.7	12.46 ± 1.9	0.13	179.74 ± 13.6
meloxicam	267.71 ± 8.1	112.67 ± 3.3	0.42	174.23 ± 20.3

**Table 15:** Results of studies of compounds **30**, **31**, and **32** obtained by Szczęśniak-Sięga.

Compound	IC_50_ (µM)		Ratio: COX-2/COX-1
	COX-1	COX-2	
**Xa**	91.2 (0.18)	54.6 (0.06)	0.60
**Xb**	89.0 (0.16)	55.9 (0.08)	0.63
**Xc**	89.3 (0.09)	55.3 (0.10)	0.62
**30**	66.1 (0.08)	56.9 (0.13)	0.86
**31a**	105.5 (0.15)	89.9 (0.14)	0.85
**31b**	86.9 (0.15)	95.1(0.10)	1.09
**31c**	89.8 (0.14)	94.1 (0.12)	1.05
**31d**	125.7 (0.07)	92.9 (0.09)	0.74
**31e**	115.3 (0.11)	56.9 (0.06)	0.49
**32**	86.1 (0.08)	54.0 (0.09)	0.63
meloxicam	83.7 (0.10)	59.2 (0.12)	0.71

SD values are given in brackets.

**Table 16:** Results of studies of compounds **35** obtained from Wang’s first modification.

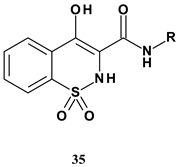			
**Compound**	**R**	**mPGES-1** **IC_50_ (µM)**	**mPGES-1/COX-2 Selectivity**
**35a**	4-(4-chlorobiphenyl)	0.29	23
**35b**	4-(4-chlorophenoxy)phenyl	0.53	10
**35c**	*N*-(4-chlorophenylo)-4-aniline	0.15	>23
**35d**	4-(4-chlorobenzoyl)-3-chlorophenyl	0.11	84
**35e**	3-(4-chlorobenzoyl)phenyl	3.91	44

**Table 17:** Results of studies of compounds **36** obtained from Wang’s second modification.

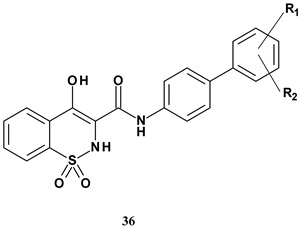				
**Compound**	**R_1_**	**R_2_**	**mPGES-1** **IC_50_ (µM)**	**mPGES-1/COX-2 Selectivity**
**36a**	H	3-Cl	0.86	22
**36b**	H	4-CH_3_	<0.07	>45
**36c**	H	4-CN	3.96	-
**36d**	H	4-H	3.13	14
**36e**	3-Cl	4-Cl	0.016	>238
**36f**	2-Cl	3-Cl	0.038	>48
**36g**	2-Cl	4-Cl	0.043	>98
**36h**	3-OCH_3_	4-OCH_3_	32.2	>77
**36i**	3-CH_3_	4-CH_3_	<0.026	14
**36j**	2-CH_3_	3-CH_3_	1.00	-

**Table 18:** Results of mPGES-1 inhibition by compounds obtained from Wang’s third modification.

Compound	mPGES-1 IC_50_ (µM)
**37**	0.16
**38**	12.7
**39**	11.7
**40**	65.6

**Table 19:** Results of mPGES-1 inhibition by compounds obtained from Wang’s fourth modification.

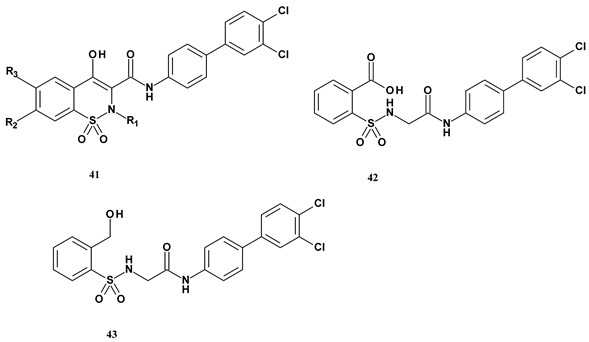					
**Compound**	**R_1_**	**R_2_**	**R_3_**	**mPGES-1** **IC_50_ (µM)**	**mPGES-1/COX-2 Selectivity**
**41a**	H	H	OCH_3_	0.69	>77
**41b**	H	H	OH	0.098	>69
**41c**	H	OCH_3_	H	3.05	-
**41d**	H	OH	H	0.18	-
**41e**	CH_3_	H	H	0.28	-
41f *	H	H	H	0.19	>149
**42**	-	-	-	5.76	>3
**43**	-	-	-	29.6	-

* This compound has a saturated ring B.

**Table 20:** Results of cytokine secretion inhibition by Gannarapu compounds from series **44** and **45**.

Compound	Secretion Inhibition Efficacy IC_50_ (µM)			Cell Viability (%)
	TNF-α	IL-8	MCP-1	
**44a**	NA	NA	NA	93.05 ± 1.09
**44b**	NA	NA	NA	91.95 ± 2.09
**44c**	NA	NA	NA	96.25 ± 1.22
**44d**	NA	NA	NA	94.23 ± 3.25
**44e**	NA	16.7 ± 1.9	5.7 ± 1.1	93.00 ± 1.26
**44f**	NA	NA	NA	97.50 ± 1.29
**44g**	NA	NA	NA	92.34 ± 2.01
**44h**	NA	NA	NA	90.25 ± 2.87
**45a**	NA	NA	NA	94.12 ± 1.69
**45b**	NA	NA	9.4 ± 0.7	95.50 ± 3.11
**45c**	NA	NA	NA	92.45 ± 1.65
**45d**	NA	NA	NA	97.50 ± 1.73
**45e**	12.1 ± 1.1	NA	4.0 ± 1.0	93.00 ± 1.41
**45f**	NA	NA	NA	95.12 ± 2.51
**45g**	NA	NA	NA	92.15 ± 1.52
**45h**	6.0 ± 0.7	NA	2.5 ± 0.9	91.25 ± 1.26
piroxicam	NA	NT	NA	-
pioglitazone	NT	NT	18.6 ± 2.1	-

NA: denotes activity >20 µM; NT: indicates “not tested”.

**Table 21:** Results of cytokine secretion inhibition by Gannarapu compounds from series **46** and **47**.

Compound		Inhibition (%)		Cell Viability
	TNF-α Secretion	IL-1β Secretion	MCP-1 Secretion	
**46b**	2.9 ± 1.2	64.7 ± 5.4	40.9 ± 6.5	92.32 ± 1.20
**46c**	24.0 ± 3.4	56.9 ± 7.8	31.9 ± 3.1	94.20 ± 3.36
**46d**	NA	50.2 ± 6.5	45.2 ± 5.3	94.32 ± 1.08
**46e**	NA	17.7 ± 2.4	12.7 ± 2.9	96.62 ± 2.42
**46f**	NA	53.9 ± 4.1	58.9 ± 6.8	94.40 ± 1.92
**46g**	NA	36.5 ± 3.7	31.5 ± 2.6	92.98 ± 2.08
**46h**	NA	43.9 ± 3.5	58.9 ± 5.6	95.32 ± 1.54
**46i**	NA	16.3 ± 6.4	NA	94.26 ± 2.60
**46j**	NA	37.3 ± 8.6	5.4 ± 1.2	96.03 ± 1.52
**46k**	NA	23.9 ± 4.5	NA	93.80 ± 3.24
**46l**	52.5 ± 6.3	61.8 ± 2.8	67.1 ± 4.5	94.26 ± 2.06
**46m**	11.2 ± 6.5	47.7 ± 3.8	29.6 ± 3.2	96.20 ± 2.30
**46n**	13.7 ± 2.9	45.9 ± 4.3	16.2 ± 3.9	94.40 ± 1.54
**46p**	48.3 ± 3.4	NA	NA	93.05 ± 2.16
**46q**	NA	42.6 ± 2.9	NA	94.15 ± 1.29
**46r**	NA	25.6 ± 4.7	30.7 ± 5.3	92.60 ± 2.20
**46s**	NA	40.2 ± 4.5	NA	96.06 ± 1.15
**47a**	33.3 ± 4.5	49.3 ± 3.2	76.7 ± 6.2	96.23 ± 1.02
**47b**	73.4 ± 6.5	42.4 ± 4.5	41.4 ± 4.5	92.5 ± 2.16
**47c**	39.7 ± 2.8	47.7 ± 5.3	56.1 ± 5.5	95.18 ± 1.19
**47d**	59.8 ± 3.8	63.8 ± 4.5	65.4 ± 3.2	93.56 ± 2.02
**47e**	94.2 ± 4.5	91.1 ± 3.2	81.1 ± 6.8	95.08 ± 1.19
**47f**	62.9 ± 5.6	49.8 ± 5.8	64.8 ± 8.2	94.45 ± 2.23
**47g**	91.1 ± 4.5	80.4 ± 6.2	84.0 ± 4.4	98.23 ± 1.45
**47h**	31.3 ± 3.2	46.3 ± 4.6	41.3 ± 2.8	95.62 ± 2.08
**47i**	84.7 ± 6.2	74.9 ± 7.2	87.2 ± 5.3	97.03 ± 1.25
**47j**	70.7 ± 5.6	69.5 ± 6.5	48.2 ± 4.8	96.45 ± 2.22
**47k**	93.2 ± 2.8	93.6 ± 4.8	88.4 ± 3.6	94.28 ± 2.04
**47l**	87.6 ± 4.8	83.6 ± 5.6	85.1 ± 5.3	96.20 ± 3.03
celecoxib	37.4 ± 8.3	31.1 ± 3.2	20.1 ± 3.2	-
piroxicam	25.8 ± 4.5	21.8 ± 6.2	11.4 ± 2.3	-

NA—no inhibition.

**Table 22:** Results of p38α MAPK and TNF-α inhibition by Sabatini compounds **48–53**.

Compound	Inhibition (%) p38α	IC_50_ (µM)		Compound	Inhibition (%) p38α	IC_50_ (µM)	
		p38α	TNF-α			p38α	TNF-α
**48a**	26.4	NC	92.3 ± 5.6	**49g**	26.6	NC	25 ± 2.1
**48b**	52.0	NC	>100	**49h**	15.0	NC	>100
**48c**	46.8	NC	>100	**49i**	28.2	NC	>100
**48d**	91.0	0.936 ± 0.296	18 ± 3.5	**49j**	25.5	NC	>100
**48e**	24.5	NC	>100	**49k**	33.9	NC	>100
**48f**	74.7	0.967 ± 0.137	>100	**50a**	65.8	7.727 ± 1.196	>100
**48g**	41.3	NC	>100	**50b**	79.5	2.863 ± 0.169	52 ± 2.5
**48h**	60.1	NC	>100	**50c**	87.0	1.949 ± 0.314	43 ± 2.8
**48i**	67.8	2.784 ± 0.694	>100	**50f**	75.3	3.332 ± 0.168	>100
**48j**	30.0	NC	>100	**50g**	51.5	NC	82.7 ± 5.2
**48k**	85.1	0.593 ± 0.099	20 ± 2.6	**50h**	78.8	2.749 ± 0.294	>100
**49a**	14.4	NC	>100	**50i**	76.5	3.529 ± 0.119	51 ± 3.2
**49b**	19.5	NC	>100	**50j**	50.0	NC	>100
**49c**	36.4	NC	>100	**50k**	81.7	1.124 ± 0.357	32.4 ± 2.8
**49d**	27.7	NC	31 ± 1.8	**51**	96	0.431 ± 0.053	>100
**49e**	35.6	NC	>100	**52**	82	2.424 ± 0.154	12 ± 1.8
**49f**	16.8	NC	>100	**53**	95	0.457 ± 0.054	0.5 ± 0.1

NC—not calculated.

## Data Availability

No new data were created or analyzed in this study. Data sharing is not applicable to this article.

## Abbreviations

The following abbreviations are used in this manuscript:

**BBB**—blood–brain barrier	**NAH**—*N*-acylhydrazone
**11β-HSD1**—11β-hydroxysteroid dehydrogenase type 1	**NHDF**—normal human dermal fibroblasts
**CBX**—carbenoxolone	**NO**—nitric oxide
**COX**—cyclooxygenase	**PCA**—passive cutaneous anaphylaxis
**ED_50_**—effective dose for 50% of the population	**PEC**—peritoneal exudate cells (rat)
**ERK**—extracellular signal-regulated kinase	**PGD_2_**—prostaglandin D_2_
**ERK5**—extracellular signal-regulated kinase 5	**PGE_2_**—prostaglandin E_2_
**FHA**—fast halo assay	**PGF_2α_**—prostaglandin F_2_ alpha
**HO-1**—heme oxygenase-1	**PGG_2_**—prostaglandin G_2_
**ID_50_**—inhibitory dose for 50% of the response	**PGH_2_**—prostaglandin H_2_
**IL-1β**—interleukin 1 beta	**PGI_2_**—prostacyclin (prostaglandin I_2_)
**IL-6**—interleukin 6	**PMA**—phorbol 12-myristate 13-acetate
**IL-8**—interleukin 8	**COPD**—chronic obstructive pulmonary disease
**JNK**—c-Jun *N*-terminal kinase	**RF**—rheumatoid factor
**LPS**—lipopolysaccharide	**ROS**—reactive oxygen species
**MAPK**—mitogen-activated protein kinase	**RA**—rheumatoid arthritis
**MCP-1**—monocyte chemoattractant protein-1	**TBA**—thiobarbituric acid
**MDA**—malondialdehyde	**TLRs**—Toll-like receptors
**MCDA**—multiple criteria decision analysis	**TNF-α**—tumor necrosis factor alpha
**mPGES-1**—microsomal prostaglandin E synthase-1	**TXA_2_**—thromboxane A_2_
**MMP**—matrix metalloproteinase	
